# A Comprehensive ^19^F NMR Framework for Fragment‐Based Drug Discovery: The Validated Screening Library OpenFL600 and Efficient Affinity Ranking by CSAR

**DOI:** 10.1002/anie.9964915

**Published:** 2026-07-23

**Authors:** Simon H. Rüdisser, Gabriela Stadler, Giacomo Padroni, Daria Barbash, Marco M. Ruckstuhl, Nils Lorz, Christina Jordan, Sarah Skibinski, Timo T. Stühlinger, Sarah E. Kratzwald, Katharina M. Siess, Sven Brüschweiler, Gerald Platzer, Antoine Cléry, Robert Konrat, Frédéric H.‐.T. Allain, Alvar D. Gossert

**Affiliations:** ^1^ Department of Biology ETH Zürich Zürich Switzerland; ^2^ Present address: Department of Chemistry and Applied Biosciences ETH Zürich Zürich Switzerland; ^3^ Present address: CPI Healthcare Glasgow United Kingdom; ^4^ Mag‐Lab Vienna Austria; ^5^ Present address: CIC bioGUNE, Derio Bizkaia Spain; ^6^ Resonate Bio Vienna Austria; ^7^ Department of Structural and Computational Biology University of Vienna Vienna Austria

**Keywords:** chemical shielding anisotropy, 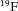
 NMR, fragment screening libraries, fragment‐based drug discovery, ligand affinity

## Abstract

Small‐molecule drug discovery relies on the identification of initial chemical starting points, or screening hits, and their subsequent optimization for potency and selectivity. Fragment‐based drug discovery (FBDD), particularly through 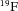 NMR screening, is a powerful method for hit identification due to its high sensitivity. Here, we present the design and validation of a non‐proprietary 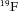 NMR screening library (OpenFL600) capable of probing diverse binding pockets, as demonstrated for RNA, G protein‐coupled receptors (GPCRs), kinases, protein–protein interactions, and proteases. Our screening campaigns yielded specific hits for 11 respective targets and therefore valuable structure–activity relationship (SAR). The absence of promiscuous binders confirms the high quality of the OpenFL600 library, a result of both careful fragment selection and rigorous molecular quality control. Subsequent affinity ranking of identified hits—typically a laborious task—is considerably streamlined by fast chemical shift anisotropy (CSA) Affinity Ranking (CSAR). CSAR is enabled by providing CSA values for each fragment. This creates a highly efficient workflow, from initial hit screening to the prioritization of leads. In summary, we provide a comprehensive framework for 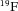 NMR‐based screening across diverse target classes, assessing their druggability, and enabling efficient affinity ranking to facilitate hit‐to‐lead progression.

## Graphical abstract

NMR screening is a powerful method for hit detection in drug‐discovery. We designed and validated the OpenFL600  NMR library to probe diverse targets, including RNA, GPCRs, kinases, and proteases. This library yields target‐specific ligands without generating promiscuous binders. Efficient affinity ranking of hits, based on chemical shift anisotropy relaxation, enables the establishment of structure–activity relationships. Our integrated framework thus supports  NMR screening, druggability assessment, and accurate affinity ranking.

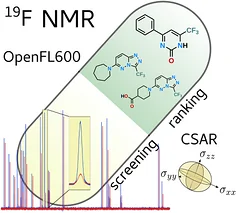

## 1 Introduction

Target‐based drug discovery relies on the identification of chemical starting points through screening campaigns. Traditionally, high‐throughput screening (HTS) of millions of molecules yields hits, which are subsequently developed into leads, drug candidates, and, ultimately, drugs [1]. Current technologies, such as DNA‐encoded libraries (DEL), enable the screening of even larger molecular collections, consisting of billions of compounds [2]. As an alternative approach, FBDD focuses on discovering small, weakly binding ligands with high ligand efficiency [3, 4]. These highly efficient fragments are then optimized into potent ligands through fragment linking, fragment growing, or fragment merging, often supported by structure‐guided drug design [5]. FBDD does explore the chemical space more exhaustively than screening larger sets of more complex molecules by HTS. Fragment screening is typically conducted using biophysical, label‐free methods, which are well‐suited to detect weak interactions. While fluorescence‐based enzymatic assays can also reliably screen fragments [6], biophysical techniques such as NMR, surface plasmon resonance (SPR) and X‐ray crystallography are less prone to readout interference, particularly at the high compound concentrations required for fragment screening [7, 8, 9, 10].


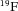
 NMR experiments are among the most sensitive techniques for binding detection [11]. Fragment screening by 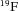 NMR ligand observation by utilizing a library of fragments with different local environment of fluorine (LEF) has been pioneered by the team at Novartis et al. [12]. Designing a fragment screening library is a critical step in FBDD. The primary goal is to ensure the library is chemically diverse and synthetically expandable, enabling the identification and optimization of novel chemical entities [13, 14, 15]. A well‐designed fragment library should encompass a broad chemical space, incorporating fragments that represent a wide range of chemical structures and pharmacophores [16]. Key properties such as purity, structural integrity and aggregation propensity must also be carefully considered. Fragments should exhibit sufficient solubility under assay conditions and be free of reactive or unstable functional groups that could interfere with screening outcomes.

The diversity of a library should ensure that ligands against a wide variety of targets can be found. While proteins remain the most common drug targets, RNA has increasingly emerged as a focus of drug discovery efforts. Historically, small molecules targeting RNA have primarily been directed at antibiotic targets, such as the prokaryotic ribosome [17]. Recent advances have highlighted RNA splicing modifiers as a promising therapeutic strategy [18, 19]. These small molecules target specific motifs in pre‐mRNA to modulate gene expression, offering potential treatments for a range of conditions, including spinal muscular atrophy (SMA). Notably, risdiplam, an FDA‐approved splicing modifier, exemplifies this approach for SMA treatment [18, 19]. Similar to protein‐targeted drug discovery, RNA has also been explored through fragment‐based drug discovery (FBDD), such that a new library should also be tested for targeting RNAs. For a comprehensive overview of FBDD applications to RNA, we refer readers to an excellent and recent review by Lundquist et al. [20].

In this work, we describe the design and preparation of chemically diverse fragment libraries comprising in total 618 molecules, tailored for screening by 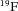 NMR. We provide a detailed account of the library design, the composition of the screening mixtures, the 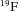 chemical shifts as well as the solubilities of the constituent fragments. To the best of our knowledge, this is the first disclosure of such comprehensive information for a publicly available 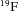 NMR screening library. We present the results of screening campaigns against a diverse set of targets, such as transferases, proteases, RNA‐binding proteins, GPCRs, and RNA. The observed hit rates range from 1% to 9%, which we interpret as a proxy for target druggability. A detailed analysis of the hit compounds enabled the identification of chemical features unique to specific binding pockets. Furthermore, we discuss the chemical and target space coverage of our library and its sub‐libraries, CF and ${\mathrm{CF}}_{3}$, along with strategies for its future expansion. The successful screening results experimentally validate the library's utility for probing diverse binding sites. We therefore present the OpenFL600 library as a validated and readily applicable screening set for fragment‐based drug discovery.

Affinity ranking of hits is invaluable for prioritizing hits from NMR‐based screens. The relative affinity data it provides enable the establishment of structure–activity relationships (SAR), which are crucial for guiding medicinal chemistry efforts, particularly in the absence of structural data for the target–ligand complex.

In order to determine the relative affinities of 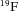 screening hits, we have previously introduced the CSAR (chemical shift‐anisotropy‐based affinity ranking) method [21]. Affinity ranking is essential to prioritize hits for hit‐to‐lead progression. CSAR allows the ranking of ligands based on 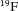 $R_{\text{2,CSA}}$ relaxation data without the need for titration experiments or for isotopic labeling of the receptor [21]. Briefly, the CSAR method consists of three steps: (1) The chemical shift anisotropy (CSA) contribution to $R_{2}$, $R_{\text{2,CSA}}$, of 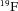 ligands is measured by separating it from the exchange and dipole‐dipole $R_{2}$ relaxation. (2) The theoretical $R_{\text{2,CSA}}$ of the free ligand is calculated using DFT methods. (3) The ratio of measured to calculated $R_{\text{2,CSA}}$ values is proportional to the fraction of bound ligand and therefore provides an accurate affinity ranking for ligands binding to the same receptor. With CSAKD [22], a refinement was recently introduced, taking into account the diffusion tensor of the target protein and the order parameter of the ligand to achieve a more precise description of $R_{\text{2,CSA}}$ in the bound state, allowing determination of the dissociation constant $K_{D}$.

**FIGURE 1:**
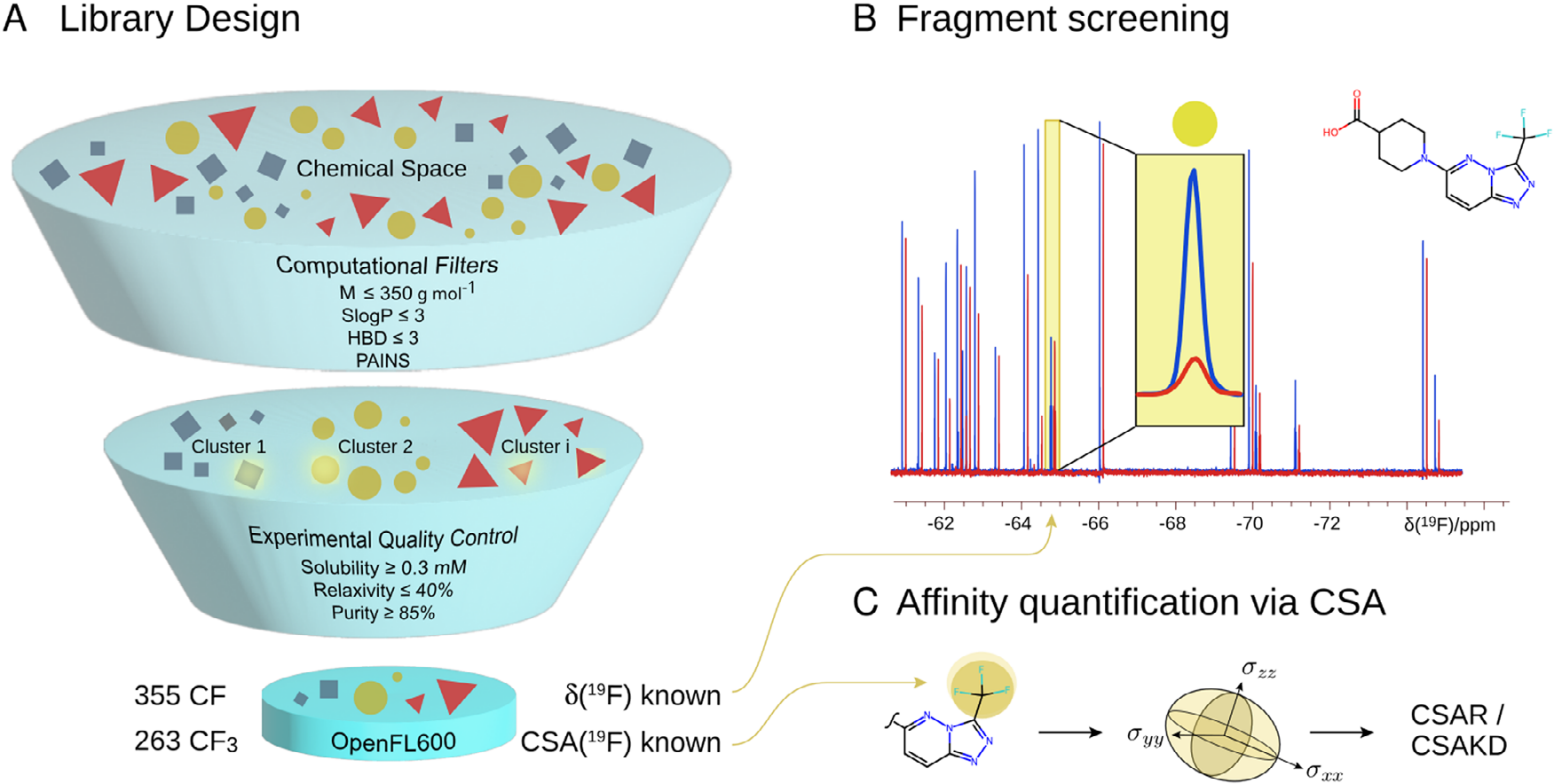
(A) A set of chemically diverse small molecules, that is, fragments, was selected from vendor catalogs using computational filters for molar mass (M), calculated partition coefficients (SlogP), number of hydrogen bond donors (HBD), and reactivity (PAINS filter [23]). From the resulting molecules, clusters of similar molecules were identified, and a representative subset from each cluster was chosen, resulting in 1207 fragments, 780 containing CF and 427 ${\mathrm{CF}}_{3}$. Each of these molecules was subsequently subjected to rigorous experimental quality control in aqueous buffer. Only molecules which met the consistency, purity, solubility, and aggregation criteria were kept (see Table 1 and main text), resulting in a total of 618 fragments for the ${\mathrm{CF}}_{3}$ and CF library. (B) The molecules were screened in mixtures (12 mixtures for CF and 12 for ${\mathrm{CF}}_{3}$) by ligand observation 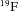 $R_{2}$ relaxation NMR experiments with a relaxation delay of 350 ms for ${\mathrm{CF}}_{3}$‐ and 200 ms for CF‐containing molecules. Interaction with the target is indicated by a decrease of the 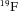 signal intensity in the presence of the receptor (red curve) compared to a sample containing the small molecules only (blue curve). The NMR data are shown for mixture nr. 2 of the ${\mathrm{CF}}_{3}$ library in the presence and absence of the 115.3 kDa homohexamer PPAT. (C) The hits found in screening can seamlessly be ranked by affinity using the CSAR [21] or CSAKD [22] approaches. This requires knowledge of the 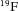 CSA, which has been calculated beforehand using DFT or machine‐learning methods for all molecules in the library.

We demonstrate the integration of the CSAR affinity‐ranking approaches into a standard workflow for 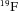 NMR screening, validation, and hit assessment across several targets. A prerequisite for calculating the $R_{\text{2,CSA}}$ relaxation rate in CSAR is knowledge of the CSA tensor of the ligand (see point 2 above). This typically requires density functional theory (DFT) calculations of several hours per ligand on super‐computing infrastructure, and expert knowledge in DFT. To make CSAR readily accessible without extensive computing resources, we provide the pre‐calculated CSA values for all molecules in the OpenFL600 screening library, obtained from extensive DFT calculations. By combining library design, NMR screening, CSA calculation, and affinity ranking, our integrated workflow streamlines fragment‐based screening and affinity ranking via 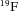 NMR for early drug discovery. An overview of this process is provided in Figure 1.

## 2 Results and Discussion

### 2.1 Design of the OpenFL600 NMR Screening Library

A set of chemically diverse molecules was assembled for NMR screening. The high diversity of molecules increases the probability that ligands bind to pockets with different characteristics, that is, for various types of enzymes such as kinases and proteases, protein–protein interaction sites, transmembrane receptors, and RNA targets [15]. Different approaches have been described to select diverse sets of molecules for screening [24, 25]. Here, we have used the clustering method, which will be described in the following.

#### 2.1.1 Molecule Selection Workflow

Molecules were selected from two vendors, Otava and Enamine, both of which provide screening sets of molecules, which contain 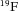. The majority of compounds were sourced from Otava, supplemented with molecules from Enamine to enhance chemical diversity. Molecules were subjected to the following filters: molar mass (M) ≤ 350 g ${\mathrm{mol}}^{-1}$, calculated partition coefficient between n‐octanol and water (SlogP) ≤ 3, and ≤ 3 hydrogen bond donors (HBD) (Figure 1A). These criteria align with the rule of three for fragments [26], where an upper M limit of 350 g ${\mathrm{mol}}^{-1}$ was chosen to account for the extra mass that a ${\mathrm{CF}}_{3}$ group adds. In addition, PAINS filters [23] (Pan Assay Interference Compounds) were applied to eliminate unstable or reactive molecules. Molecules containing two or more inequivalent fluorine atoms were excluded from the library, since $J_{\mathrm{FF}}$ scalar couplings are very strong and visible even across several bonds. Presence of more than one fluorine atom typically leads to peak splitting, which in turn yields lower intensity of the individual signals and twisted phases in spin echo experiments.

To optimize diversity, a distance matrix was subsequently calculated based on the size of the maximum common substructure (MCSS) [27] for 665 ${\mathrm{CF}}_{3}$ containing molecules and 1164 molecules with 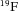 connected to an aromatic ring (CF‐molecules). To group the molecules into structurally similar sets, *k*‐medoids clustering was performed [28]. The number of clusters and their sizes were optimized to maximize diversity within each cluster and ensure comprehensive coverage of chemical space. The workflow for the selection of CF and ${\mathrm{CF}}_{3}$ fragments is illustrated in Supporting Information Figures S1 and S2. The final selection comprised 32 ${\mathrm{CF}}_{3}$ clusters and 35 CF clusters, containing 22 ${\mathrm{CF}}_{3}$ and 20 CF molecules per cluster, respectively. This resulted in 780 CF and 427 ${\mathrm{CF}}_{3}$ molecules for the screening sets. In total, 1207 molecules have been ordered and tested by 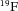 and 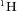 NMR as detailed in the next section.

#### 2.1.2 Solubility, Purity, Aggregation Properties and Mixture Preparation

The selected molecules for the CF and ${\mathrm{CF}}_{3}$ libraries were analyzed using 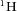 NMR to assess (1) agreement between the NMR spectrum and the molecular structure, (2) solubility, and (3) purity (see legend of Table 1 for cut‐off values). Additionally, 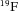 $T_{2}$‐relaxation NMR data were utilized to identify molecules prone to aggregation. Such molecules were excluded from the screening set to minimize the risk of co‐aggregation with other compounds in the mixtures, which could lead to false‐positive results. Only molecules that met the defined quality criteria were included in the screening library. This results in a screening set of 263 ${\mathrm{CF}}_{3}$ and 355 CF molecules (Figure 1A). The measured 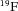 chemical shifts were used to group molecules into mixtures, ensuring no signal overlap. Mixtures were prepared separately for ${\mathrm{CF}}_{3}$ and CF molecules due to differences in their testing concentrations, which are typically 30 μM for ${\mathrm{CF}}_{3}$ and 40 μM for CF. Additionally, considering the broad dispersion of 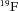 chemical shifts and the distinct chemical shift ranges of ${\mathrm{CF}}_{3}$ and CF signals it may be convenient to use different spectral widths and carrier offsets during data acquisition. Furthermore, because of the generally large CSA of CF molecules, which results in faster relaxation, the relaxation delay for $T_{2}$‐screening was set to 200 ms. ${\mathrm{CF}}_{3}$ molecules with an on average lower CSA require a longer $T_{2}$ relaxation delay of typically 350 ms. The calculated CSA for both sets, expressed as the average $\delta_{\sigma }$ and η values are provided in Table 1. For the CF library, 28–30 molecules were combined into each of the 12 mixtures, while for the ${\mathrm{CF}}_{3}$ library, 20–24 molecules were combined into each of the 12 mixtures (Figure 1B).

**TABLE 1:** The number of molecules selected for the screening libraries, along with their average molecular properties, is presented.

	${\mathrm{CF}}_{3}$	CF
Tested by 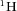 , 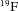 NMR	427	780
Structure consistent, < 15% impure	419	648
Soluble up to 0.3 mM	287	506
$T_{2}$ filter: > 60% intensity	317	460
nr. of molecules in screening set	263	355
nr. of mixtures	12	12
average M/g mol^−1^	256.5	227.0
average SlogP	2.02	1.87
average nr. of HBD	1.1	1.0
average nr. of HBA	3.2	2.7
average nr. of rotatable bonds	1.9	2.0
fraction sp^3^ C atoms	0.38	0.24
average $\delta_{\sigma }$/ppm	43.52	69.53
average η/ppm		0.998

*Note*: Compounds were evaluated for solubility, purity, and aggregation using 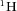 and 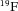 NMR prior to their inclusion in mixtures. The selection criteria were as follows: (1) the 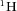 NMR spectrum must be consistent with the molecular structure, (2) solubility must exceed 0.3 mM, (3) impurities must constitute less than 15%, and (4) more than 60% of the 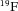 signal intensity must remain after applying a $T_{2}$ relaxation filter delay of 350 ms for ${\mathrm{CF}}_{3}$ groups and 200 ms for CF groups. The final set of molecules selected for screening represents the intersection of all compounds meeting the aforementioned criteria. For a subset of the library, the reduced anisotropy ($\delta_{\sigma }$) and axial asymmetry (η) of the 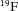 CSA tensor were calculated using DFT methods, as detailed in the Supporting Information. These parameters were determined for 326 CF‐containing and 181 ${\mathrm{CF}}_{3}$‐containing molecules.

### 2.2 Validation of OpenFL600 Library by Screening of Protein and RNA Targets

#### 2.2.1 Molecular Properties of Ligands

The CF and ${\mathrm{CF}}_{3}$ libraries have been screened against several protein and RNA targets (see Table 2). The targets include enzymes, protein protein interactions (PPIs), RNA‐binding motifs, and a trans‐membrane receptor. Ligands were identified for all targets, with $K_{D}$ values ranging from 400 μM to 7 mM. The hit rates, which reflect the presence and specific features of binding pockets for small molecules (i.e., druggability) [29], varied significantly across targets, ranging from 1% up to 9%.

**TABLE 2:** Hits from ligand screening by NMR are shown for several protein and two RNA targets.

Target	Target Class	M/10^3^ g mol^−1^	library	n(hits)	$K_{D}$ range / μM
M^pro^	Protease	2 × 34.9	${\mathrm{CF}}_{3}$	18 (10^#^)	3000 – 7000
PPAT (CoaD)	Transferase	6 × 19.2	${\mathrm{CF}}_{3}$	13 (11^#^)	400 – 500
SARS‐CoV‐2 N‐CTD	RNA‐binding, PPI	18.0	${\mathrm{CF}}_{3}$	8 (1^#^)	3000
SRSF1‐RRM12	RNA‐binding	21.7	${\mathrm{CF}}_{3}$, CF	6 (5^|^, 1^#^)	
WDR5	PPI	5 × 34.2	${\mathrm{CF}}_{3}$, CF	24 (12^#^)	
$\beta_{1}$‐adrenergic receptor	Transmembrane receptor	> $56.6^{⊥}$	CF	72 (37^*^)	
FKBP12	isomerase	12.0	${\mathrm{CF}}_{3}$, CF	6 (4^#^)	200 – 800
U1 snRNA SL3	RNA	28 nt, 9.0	${\mathrm{CF}}_{3}$	2 (2^#^)	560
s2m 3 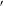 ‐UTR	RNA	45 nt, 14.6	${\mathrm{CF}}_{3}$	3 (3^#^)	1500
LecA	Carbohydrate‐binding	2 × 12.8	${\mathrm{CF}}_{3}$, CF	10 (6^||^)	
LecB	Carbohydrate‐binding	4 × 11.7	${\mathrm{CF}}_{3}$, CF	3 (2^##^)	

*Note*: Binding of the small molecule is identified by an increase in $R_{2}$ relaxation in the presence of the receptor. The initial hits were subsequently validated by competition experiments with a high affinity ligand or by target observation experiments utilizing isotopically labeled protein or unlabeled RNA. The number of validated hits is given in brackets. For targets with limited supply, only one of the libraries was screened, where, in hindsight, the CF library generally resulted in a higher hit rate.

^#^Specific binding confirmed by target observation. Competitive binding with ^|^ RNA UCAUUGGAU, ^*^ pindolol, ^||^ β‐phenyl‐galactopyranose, ^##^ fucose, 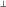 screened in presence of detergent micelles, incl. counterscreen. nt …nucleotides

**FIGURE 2:**
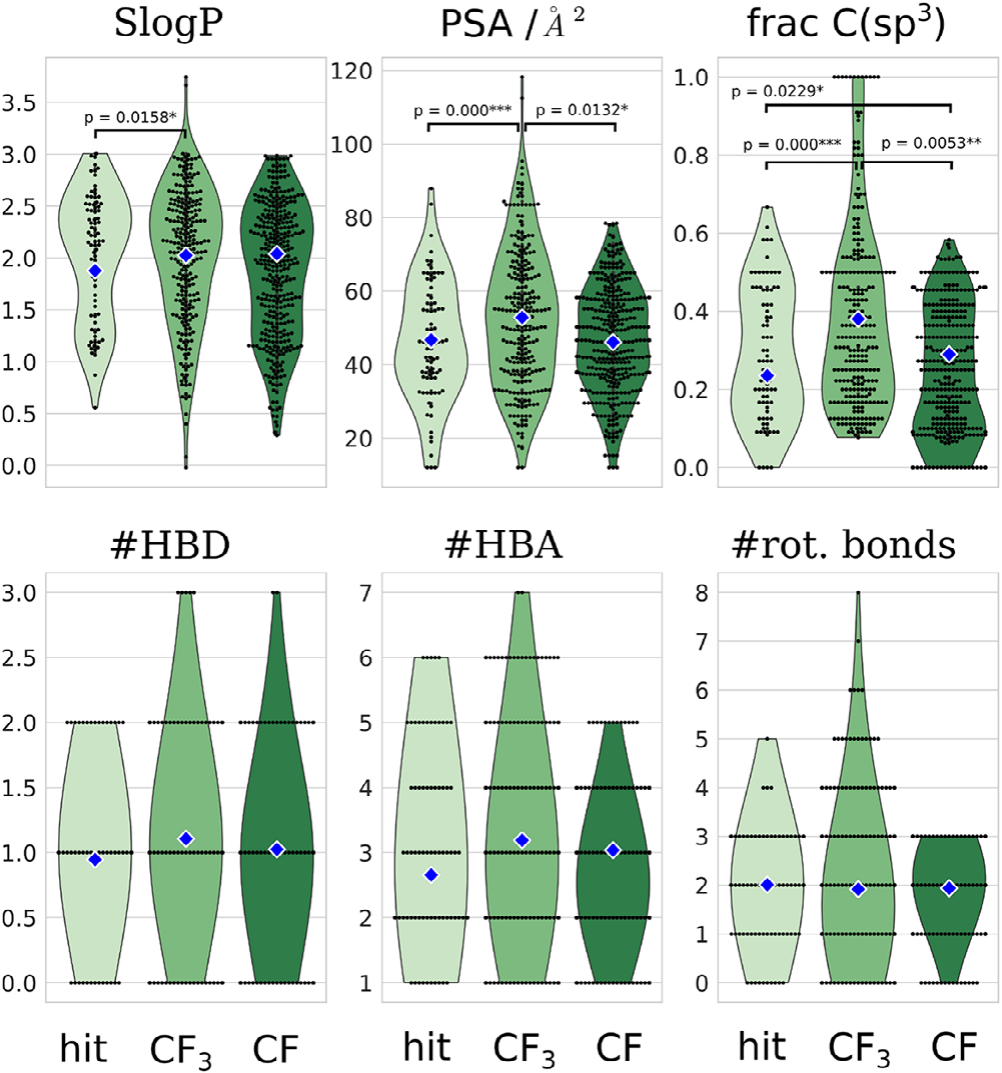
The molecular properties of the CF library, the ${\mathrm{CF}}_{3}$ library and all 91 confirmed hits from Table 2 are shown. SlogP is the predicted octanol–water partition coefficient [33], PSA is the polar surface area, or topological polar surface area [34]. The significance of differences in SlogP, PSA, and the fraction of ${\mathrm{sp}}^{3}$ carbon atoms between libraries was assessed using Student's *t*‐test, with p‐values < 0.05, 0.01, and 0.001 considered statistically significant (*), very significant (**), and highly significant (***), respectively. The ${\mathrm{CF}}_{3}$ set is enriched with polar molecules (i.e., higher PSA and lower SlogP) compared to the hits. The set of ${\mathrm{CF}}_{3}$ contains more flexible molecules compared to CF and the hits, as indicated by a higher fraction of sp^3^ carbon atoms and the increased number of rotatable bonds.

For a detailed comparison of the screening sets and the hits, relevant molecular properties were calculated. While numerous properties have been reported in the literature [30], we focused on a small subset commonly used to describe the binding features of small molecules. These include the calculated partition coefficient between n‐octanol and water (SlogP), the number of hydrogen bond donors (HBD) and acceptors (HBA), the polar surface area (PSA), the fraction of sp^3^ atoms, and the number of rotatable bonds [31]. Note that for this analysis, the C–${\mathrm{CF}}_{3}$ bond does not count as rotatable bond and that 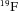 is not counted as HBA. These properties can be readily calculated from the chemical structure of the small molecule using the RDKit molecular descriptors module [32]. The molecular features for the two screening libraries, CF and ${\mathrm{CF}}_{3}$, as well as for the aggregated hits, are shown in Figure 2. Notably, the fraction of sp^3^ carbon atoms, the number of rotatable bonds, and the number of HBA are lower for the CF‐containing library compared to the ${\mathrm{CF}}_{3}$‐containing library. On average, the aggregated hits show a lower number of HBA and a lower fraction of ${\mathrm{sp}}^{3}$ carbon atoms compared to the CF or ${\mathrm{CF}}_{3}$ set (see Figure 2). Furthermore, only 5 out of 92 hits (5.4%) have more than three rotatable bonds. In the ${\mathrm{CF}}_{3}$ library, 41 out of 263 molecules (15.6%) possess more than three rotatable bonds, whereas none of the molecules in the CF library exceeds more than three rotatable bonds. Here, the C–${\mathrm{CF}}_{3}$ bond is not counted as rotatable. We speculate that molecules with fewer rotatable bonds and reduced flexibility interact more favorably with the target due to the lower entropic penalty associated with binding.

We observed a higher fraction of CF‐containing molecules among the hits, whereas ${\mathrm{CF}}_{3}$‐containing molecules were underrepresented. Specifically, for FKBP12, 3 out of 4 hits were CF‐containing molecules; for LecA and LecB, all hits contained CF; 92% of WDR5 hits contained CF; and 4 out of 5 N‐CTD ligands contained CF. The difference in hit rates between CF and ${\mathrm{CF}}_{3}$ molecules could be attributed to the ability of aromatic fluorine to form directed interactions within the binding pocket, thereby enhancing binding affinity [35, 36, 37]. Although the ${\mathrm{CF}}_{3}$ group can also participate in interactions, these are generally weaker due to the bulkiness of ${\mathrm{CF}}_{3}$, which may disrupt favorable contacts with the target [38, 39].

#### 2.2.2 NMR Screening Hits Show Target Specific Molecular Features

**FIGURE 3:**
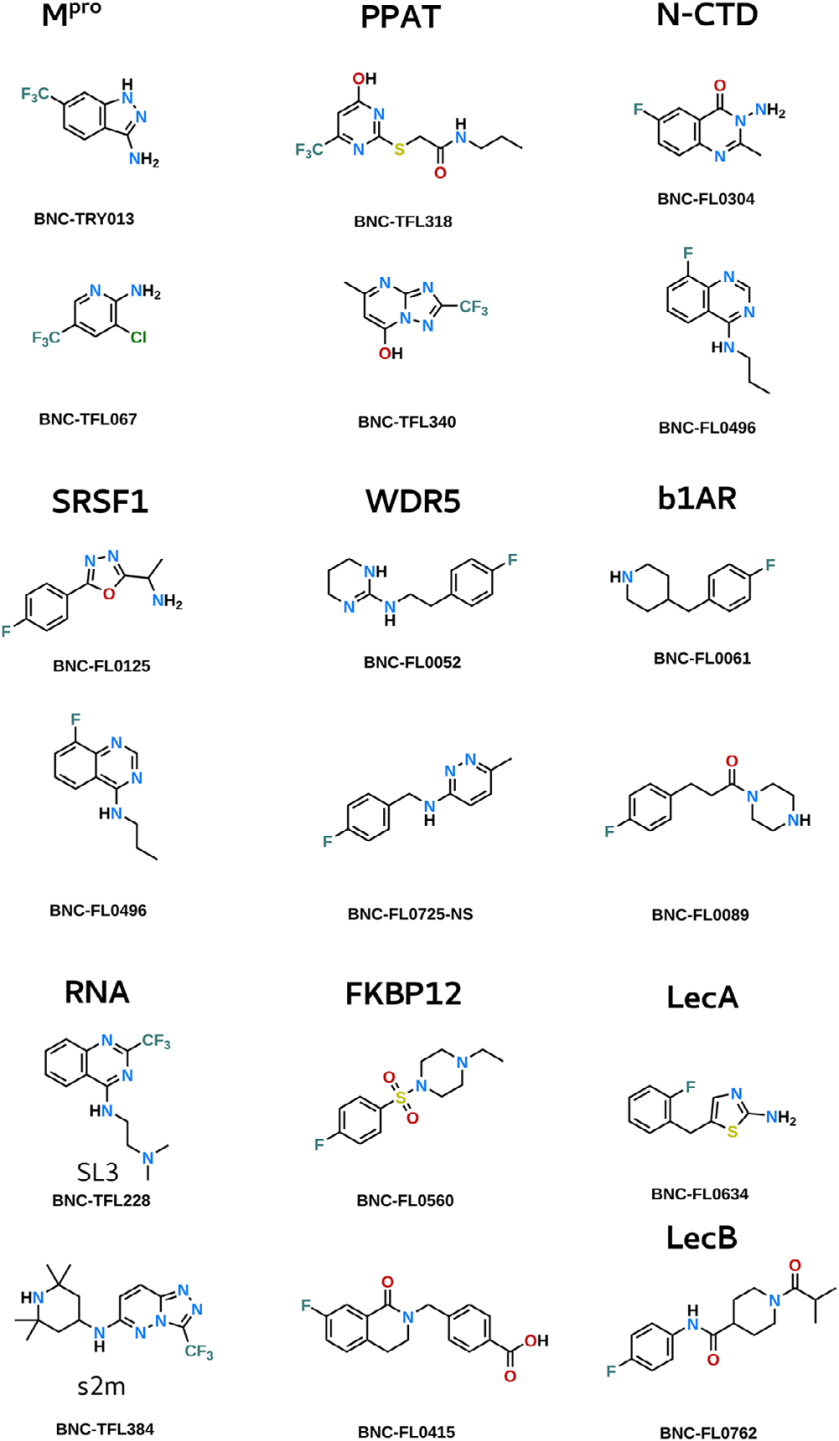
For each target, two representative 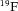 screening hits are presented. The molecule identifier is provided underneath the corresponding chemical structure.

In this section, we briefly describe the screened targets and discuss target‐specific molecular features of selected ligands (Table 2; a more extensive description can be found in the Supporting Information). The targets include enzymes such as the SARS‐CoV‐2 main protease (M^pro^), an attractive target for antiviral drug development [40], and the prolyl *cis‐trans* isomerase FKBP12, whose inhibitors have been reported to exhibit neuroprotective properties [41]. Another enzyme screened against the OpenFL600 library was phosphopantetheine adenylyltransferase (PPAT, CoaD), an essential enzyme in coenzyme A (CoA) biosynthesis that catalyzes the conversion of phosphopantetheine to dephospho‐coenzyme A. Owing to its critical role in bacterial metabolism, PPAT represents a promising target for antibacterial drug discovery [42]. A recent study from Novartis demonstrated the successful application of fragment‐based drug discovery to identify PPAT inhibitors [43]. Notably, compound 30 reported in that study exhibits high structural similarity to BNC‐TFL340 (see Figure 3), a ligand identified in the present work.

Among the screened targets were two proteins involved in protein–protein interactions: WD repeat domain 5 (WDR5) and the C‐terminal domain (CTD) of the SARS‐CoV‐2 nucleocapsid (N) protein. WDR5 is a member of the WD40‐repeat protein family and plays a central role in mediating protein–protein interactions within several chromatin‐regulating complexes [44]. The SARS‐CoV‐2 N‐CTD constitutes the C‐terminal domain of the viral nucleocapsid protein and is involved in oligomerization as well as viral RNA packaging [45, 46].

To evaluate the OpenFL600 library for RNA binding proteins, we screened the RNA recognition motifs RRM1 and RRM2, subdomains of the serine and arginine splicing factor 1 (SRSF1). SRSF1 is a member of the serine/arginine rich (SR) protein family and plays a crucial role in RNA metabolism, including pre‐mRNA splicing, mRNA stability, and translation [47, 48, 49].

The $\beta_{1}$‐adrenergic receptor ($\beta_{1}\mathrm{AR}$) was chosen as a representative of membrane proteins, as G‐protein‐coupled receptor (GPCR) drug discovery remains a major focus in pharmaceutical research. $\beta_{1}\mathrm{AR}$ is predominantly expressed in cardiac tissue and plays a vital role in regulating blood pressure homeostasis [50].

Targeting RNA represents an alternative mode of action for small molecules and we have therefore included two RNA targets in our screening campaign. Stem‐loop 3 (SL3) of the U1 snRNA is known to interact with RNA binding motif 39 (RBM39) [19]. Additionally, the stem‐loop II‐like motif (s2m) is a conserved feature of the $3^{\prime }$ untranslated region of the SARS‐CoV‐2 viral genome [51]. 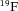 NMR screening of our library identified chemotypes similar to those described by Childs‐Disney [52] and Sreeramulu [53]: specifically, a heterocyclic aromatic system, such as triazolopyridine or quinazoline, linked by an aliphatic chain to a primary or secondary amine. This amine carries a positive charge at neutral pH and is presumed to interact with the negatively charged oxygen atoms of the RNA backbone.

The two screened carbohydrate‐binding proteins, LecA and LecB, play important roles in biofilm formation and are consequently implicated in chronic infections [54, 55]. Detailed descriptions of all targets are provided in the Supporting Information.

The hits identified were predominantly selective, with no promiscuous binders observed. Only 6 of the 91 hits interacted with 2 targets, and none bound to more than two. Some overlap among hits is expected for fragments bearing non‐specific pharmacophores, such as hydrophobic groups. For example, BNC‐FL0089, which binds to both WDR5 and $\beta_{1}\mathrm{AR}$, likely occupies a hydrophobic pocket via its *p*‐fluorophenyl moiety. Interestingly, RNA binders were specific to either SL3 or s2m. These findings highlight the high quality of the OpenFL600 library and its low propensity to yield promiscuous binders.

**FIGURE 4:**
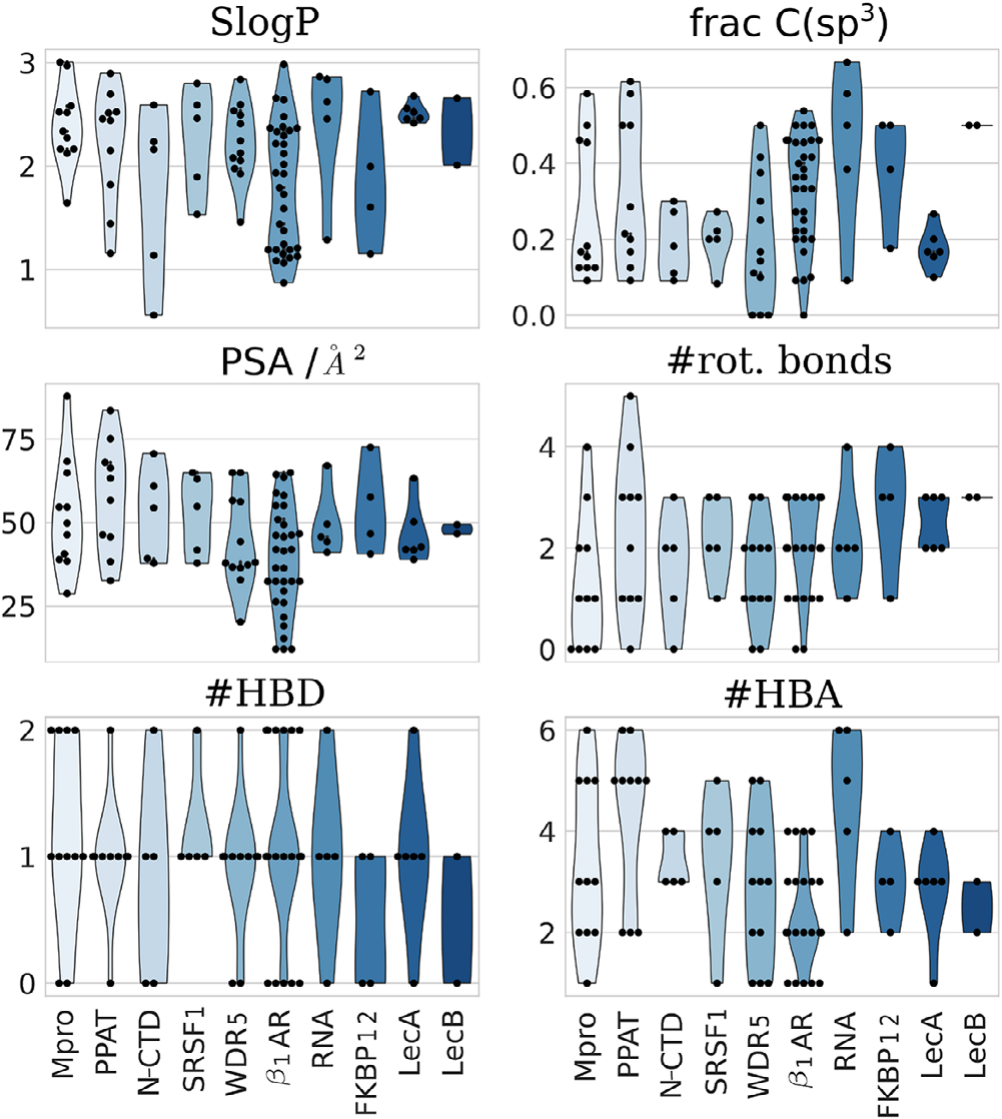
Ligands targeting individual receptors exhibit distinct molecular features that reflect the properties of their respective binding pockets. For instance, ligands targeting the $\beta_{1}$‐adrenergic receptor display the lowest PSA, suggesting a predominantly hydrophobic binding site. In this analysis, only hits validated through target observation or competitive binding experiments with known ligands were included.

The properties across all tested targets indicate some overall trends for binding propensity (Figure 2), and the molecular features of ligands for a given target are distinct and differ from those of the entire set of hits (see Figure 4). For instance, the number of HBA is slightly increased for M^pro^ ligands and more significantly enhanced for RNA and PPAT ligands. This is due to a high fraction of heterocyclic aromatic rings, particularly those containing nitrogen as an HBA, among the RNA and PPAT hits, while this feature is less prevalent in M^pro^ hits. Additionally, $\beta_{1}\mathrm{AR}$ ligands exhibit, on average, a lower SlogP and an decreased PSA compared to M^pro^ ligands, reflecting the relative hydrophobicity of their respective binding pockets.

### 2.3 Efficient Affinity Ranking of Ligands From NMR CSA Relaxation Rates

After identifying initial hits, the relative affinity of each ligand must be characterized in order establish structure–activity relationships and to subsequently prioritize hits for hit‐to‐lead activities. Here, we apply the efficient CSAR approach [21], which is briefly summarized as follows: the ranking of binding affinities is based on the difference in $R_{\text{2,CSA}}$ relaxation rates ($\Delta R_{\text{2,CSA}}$) measured at two different $B_{0}$ fields. By calculating the difference in relaxation rates at two fields, the dipolar contribution to $R_{2}$ is eliminated. Furthermore, by performing measurements under spin‐lock conditions, the exchange contribution to $R_{2}$ is also eliminated. Consequently, only the CSA contribution to $R_{2}$ remains. Because the CSA contribution for a given ligand depends on the CSA tensor and the rotational correlation time of the ligand in the bound state, the measured $\Delta R_{\text{2,CSA}}$ is scaled by the approximated, theoretical CSA rate ($\Delta R_{\text{2,CSA,calc}}$) to yield the fraction of bound ligand. We recently further refined the method by introducing CSAKD, where the relaxation rate of the ligand in the bound state is calculated with higher accuracy, such that $K_{D}$‐values can be derived [22].

A prerequisite for the CSA‐based methods is knowledge of the CSA tensor of the ligand, which we provide for the OpenFL600 library, obtained through extensive DFT calculations. In addition, the CSA values for all 1207 molecules that were tested by 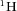 and 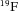 NMR for quality control are provided (Supporting Information). These CSA values were computed using a novel machine‐learning approach, which is based on molecular fingerprints and offers superior efficiency compared to DFT methods [22].

**FIGURE 5:**
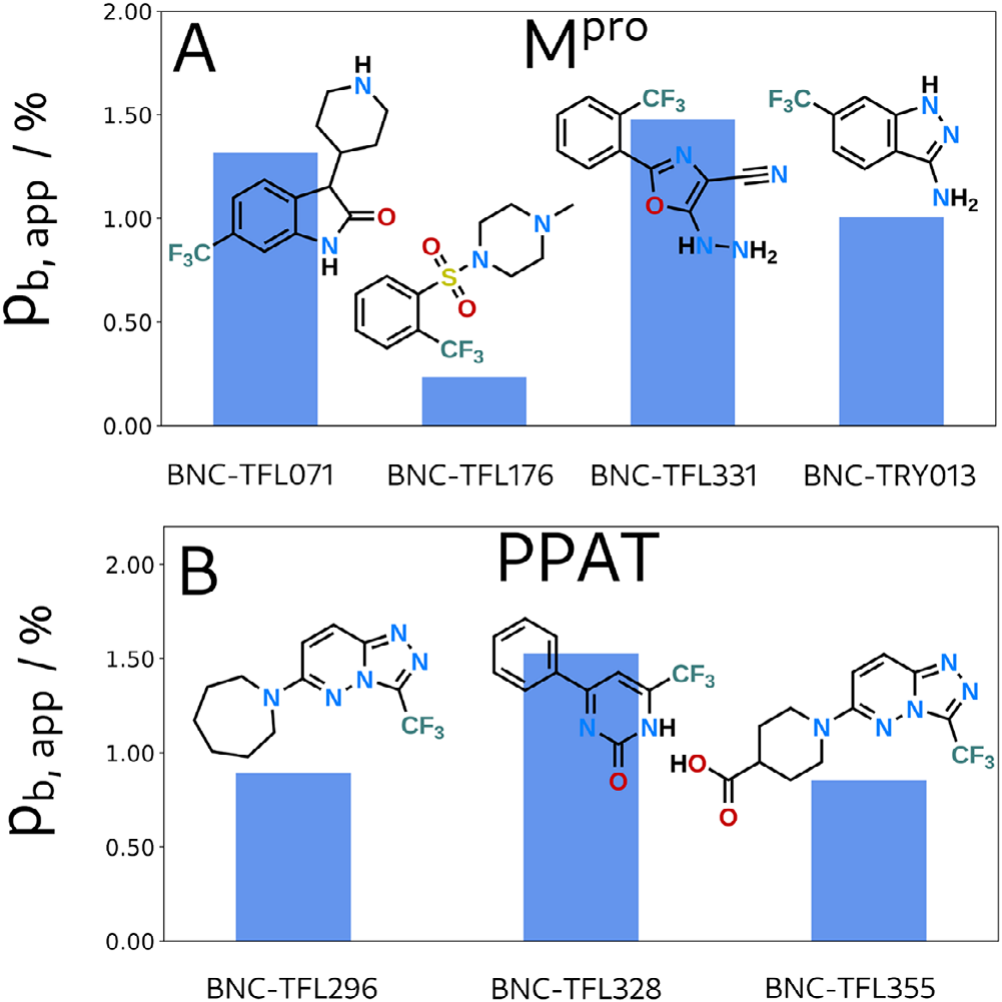
The relative affinities, expressed as the apparent fraction of bound ligand ($p_{\text{b,app}}$ in %), are shown for (A) M^pro^ and (B) PPAT. The $p_{\text{b,app}}$ values were calculated from the difference in the observed relaxation rate ($\Delta R_{\text{2,CSA}}$) measured at two magnetic fields (11.75 and 16.4 T) and the calculated relaxation rate $\Delta R_{\text{2,CSA, calc}}$ [21]. The estimated error in $p_{\text{b,app}}$ is 0.04%. $R_{\text{2,CSA}}$ rates were measured under spin‐lock and on‐resonance conditions using single‐molecule samples. A PPAT concentration of 5 μM was used, whereas the M^pro^ concentration was 30 μM with a ligand conentration of 200 μM. The $K_{D}$ values for some ligands have been determined by NMR titration experiments [22] and are provided in the following: $K_{D}$(BNC‐TFL071, M^pro^) = 3187 ± 192 μM, $K_{D}$(BNC‐TRY013, M^pro^) = 6748 ± 237 μM, $K_{D}$(BNC‐TFL355, PPAT) = 437.1 ± 66.5 μM.

Figure 5 presents the CSAR ranking for two targets, M^pro^ and PPAT. The ranking is based on the apparent fraction of bound ligand, $p_{\text{b,app}}$, defined as the ratio of the measured to the calculated difference in the $R_{2,\text{CSA}}$ relaxation rate at two distinct $B_{0}$ fields, following the CSAR methodology [21]. For two M^pro^ ligands, BNC‐TFL071 and BNC‐TRY013, whose $K_{D}$ values are known to be in the mM range, the CSAR ranking is comparable. While BNC‐TFL355 shows a $K_{D}$ value for PPAT that is approximately 10‐fold lower than those of the aforementioned M^pro^ ligands, its corresponding $p_{\text{b,app}}$ value is also lower. This observation supports the expectation that $p_{\text{b,app}}$ values are appropriate only for ranking ligands binding to the same target under identical experimental conditions, because the tumbling rate of the bound state depends on the receptor, and the fraction of bound ligand depends on the ligand and receptor concentrations.

## 3 Conclusion

We present here a 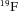 NMR fragment screening pipeline that enables hit finding and subsequent affinity ranking in one seamless process. The first element is the novel screening library OpenFL600 consisting of two screening sets: the CF and the ${\mathrm{CF}}_{3}$ libraries. We demonstrated that hits were obtained for diverse targets, validating the generality of the library. Promiscuous binders, for example, ligands that interact with more than two targets, were absent in the hit sets.

Cheminformatics analysis of the hits revealed general trends of the molecular properties as discussed in the following. Hits are enriched with hydrophobic molecules, with fewer rotatable bonds and a lower fraction of ${\mathrm{sp}}^{3}$ carbon atoms compared to the ${\mathrm{CF}}_{3}$ screening set. The properties of the aggregated hits resemble more closely the CF screening set than the ${\mathrm{CF}}_{3}$ set of molecules. As a consequence, supplementing the library with corresponding fragments is expected to increase the overall hit rate. This, however, comes with the drawback of reducing chemical diversity and, consequently, decreasing the probability of discovering ligands for targets that predominantly bind polar ligands, like carbohydrate‐binding proteins. In general, the CF library yielded a higher hit rate than the ${\mathrm{CF}}_{3}$ set, such that if target protein amounts are limiting, screening the CF library is recommended.

Based on our screening results, we propose expanding the libraries to explore additional chemical space. For instance, the hit rate for LecB, a carbohydrate‐binding protein, suggests that carbohydrate‐like molecules are underrepresented. A similar trend was observed for RNA targets, which yielded few hits but with an elevated proportion of C(${\mathrm{sp}}^{3}$) atoms. LecB and RNA hits show a high fraction of C(${\mathrm{sp}}^{3}$)‐hybridized carbon atoms, indicating that enriching the library with C(${\mathrm{sp}}^{3}$)‐containing molecules could be a promising strategy [56].

For a given target, the shared molecular properties of hits provide insight into interaction propensities, facilitating the selection of follow‐up compounds for iterative, target‐focused screening. This SAR information can be augmented by ligand epitope mapping via NMR spectroscopy [57, 58] and by analyzing the bound ligand conformation by transferred‐NOE experiments [59] to enable more informed compound selection. Additionally, the bound‐state 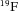 chemical shift, extracted from relaxation dispersion experiments [60], serves as a sensitive probe of the local binding pocket environment. Together, these data are invaluable for generating and evaluating protein–ligand docking hypotheses [61].

In summary, we present the design and application of a 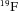 fragment screening library. The value of the library was shown through screening against a diverse set of targets, including RNA, GPCRs, protein–protein interactions, and various enzymes. The resulting hits exhibited target‐specific properties reflective of binding pocket characteristics, providing a foundation to guide SAR expansion. Furthermore, we applied the CSAR method for rapid, titration‐free affinity ranking, a critical advancement for efficient hit prioritization and SAR development. The integrated workflow, encompassing library design, screening, CSA prediction, and affinity ranking, streamlines early‐stage drug discovery efforts.

## Conflicts of Interest

The authors declare no conflicts of interest.

## Supporting information




**Supporting File 1**: anie73662‐sup‐0001‐SuppMat.pdf.

**Supporting File 2**: anie73662‐sup‐0002‐DataFiles.zip.

## Data Availability

The data that support the findings of this study are openly available in ETH research collection at https://doi.org/10.3929/ethz‐c‐000791936.

## References

[1] M. J. Wildey , A. Haunso , M. Tudor , M. Webb , and J. H. Connick , High‐Throughput Screening 50 (2017): 149–195.

[2] A. Gironda‐Martínez , E. J. Donckele , F. Samain , and D. Neri , “DNA‐Encoded Chemical Libraries: A Comprehensive Review With Succesful Stories and Future Challenges,” ACS Pharmacology & Translational Science 4, no. 4 (2021): 1265–1279. 10.1021/acsptsci.1c00118 PMC8369695 34423264

[3] P. J. Hajduk and J. Greer , “A Decade of Fragment‐Based Drug Design: Strategic Advances and Lessons Learned,” Nature Reviews Drug Discovery 6, no. 3 (2007): 211–219. 10.1038/nrd2220 17290284

[4] S. D. Bembenek , B. A. Tounge , and C. H. Reynolds , “Ligand Efficiency and Fragment‐Based Drug Discovery,” Drug Discovery Today 14, no. 5‐6 (2009): 278–283. 10.1016/j.drudis.2008.11.007 19073276

[5] C. W. Murray and T. L. Blundell , “Structural Biology in Fragment‐Based Drug Design,” Current Opinion in Structural Biology 20, no. 4 (2010): 497–507. 10.1016/j.sbi.2010.04.003 20471246

[6] A. Boettcher , S. Ruedisser , P. Erbel , et al., “Fragment‐Based Screening by Biochemical Assays,” Journal of Biomolecular Screening 15, no. 9 (2010): 1029–1041. 10.1177/1087057110380455 20855559

[7] C. Dalvit , “NMR Methods in Fragment Screening: Theory and a Comparison With Other Biophysical Techniques,” Drug Discovery Today 14, no. 21‐22 (2009): 1051–1057. 10.1016/j.drudis.2009.07.013 19716431

[8] I. Navratilova and A. L. Hopkins , “Fragment Screening by Surface Plasmon Resonance,” ACS Medicinal Chemistry Letters 1, no. 1 (2010): 44–48. 10.1021/ml900002k PMC4007845 24900174

[9] R. J. Hall , P. N. Mortenson , and C. W. Murray , “Efficient Exploration of Chemical Space by Fragment‐Based Screening,” Progress in Biophysics and Molecular Biology 116, no. 2‐3 (2014): 82–91. 10.1016/j.pbiomolbio.2014.09.007 25268064

[10] M. Pellecchia , I. Bertini , D. Cowburn , et al., “Perspectives on NMR in Drug Discovery: A Technique Comes of Age,” Nature Reviews Drug Discovery 7, no. 9 (2008): 738–745. 10.1038/nrd2606 PMC2891904 19172689

[11] C. Dalvit and A. Vulpetti , “Ligand‐Based Fluorine NMR Screening: Principles and Applications in Drug Discovery Projects,” Journal of Medicinal Chemistry 62, no. 5 (2019): 2218–2244. 10.1021/acs.jmedchem.8b01210 30295487

[12] A. Vulpetti , U. Hommel , G. Landrum , R. Lewis , and C. Dalvit , “Design and NMR‐Based Screening of LEF, a Library of Chemical Fragments With Different Local Environment of Fluorine,” Journal of the American Chemical Society 131, no. 36 (2009): 12949–12959. 10.1021/ja905207t 19702332

[13] M. Bon , A. Bilsland , J. Bower , and K. McAulay , “Fragment‐Based Drug Discovery—The Importance of High‐Quality Molecule Libraries,” Molecular Oncology 16, no. 21 (2022): 3761–3777. 10.1002/1878-0261.13277 PMC9627785 35749608

[14] W. Lau , J. Withka , D. Hepworth , et al., “Design of a Multi‐Purpose Fragment Screening Library Using Molecular Complexity and Orthogonal Diversity Metrics,” Journal of Computer‐Aided Molecular Design 25, no. 7 (2011): 621–636. 10.1007/s10822-011-9434-0 21604056

[15] A. Schuffenhauer , S. Ruedisser , A. Marzinzik , W. Jahnke , P. Selzer , and E. Jacoby , “Library Design for Fragment Based Screening,” Current Topics in Medicinal Chemistry 5, no. 8 (2005): 751–762. 10.2174/1568026054637700 16101415

[16] J. Revillo Imbernon , C. Jacquemard , G. Bret , G. Marcou , and E. Kellenberger , “Comprehensive Analysis of Commercial Fragment Libraries,” RSC Medicinal Chemistry 13, no. 3 (2021): 300–310. 10.1039/d1md00363a PMC8942207 35434627

[17] J. Thomas and P. Hergenrother , “Targeting RNA With Small Molecules,” Chemical Reviews 108, no. 4 (2008): 1171–1224. 10.1021/cr0681546 18361529

[18] S. J. Barraza , A. Bhattacharyya , C. R. Trotta , and M. G. Woll , “Targeting Strategies for Modulating pre‐mRNA Splicing With Small Molecules: Recent Advances,” Drug Discovery Today 28, no. 1 (2023): 103431. 10.1016/j.drudis.2022.103431 36356786

[19] S. Campagne , D. Jutzi , F. Malard , et al., “Molecular Basis of RNA‐Binding and Autoregulation by the Cancer‐Associated Splicing Factor RBM39,” Nature Communications 14, no. 1 (2023): 5366. 10.1038/s41467-023-40254-5 PMC10477243 37666821

[20] K. P. Lundquist , V. Panchal , C. H. Gotfredsen , R. Brenk , and M. H. Clausen , “Fragment‐Based Drug Discovery for RNA Targets,” ChemMedChem 16 (2021): 2588–2603. 10.1002/cmdc.202100324 34101375

[21] S. H. Rüdisser , N. Goldberg , M.‐O. Ebert , H. Kovacs , and A. D. Gossert , “Efficient Affinity Ranking of Fluorinated Ligands by ^19^F NMR: CSAR and FastCSAR,” Journal of Biomolecular NMR 74, no. 10‐11 (2020): 579–594. 10.1007/s10858-020-00325-x 32556806

[22] S. H. Rüdisser , G. Stadler , and A. D. Gossert , “CSAKD: Determining Absolute Ligand Affinities From ^19^F NMR Chemical Shift Anisotropy,” Angewandte Chemie International Edition (2026): e6036832, 10.1002/anie.6036832. PMC13452527 42283365

[23] J. B. Baell and G. A. Holloway , “New Substructure Filters for Removal of Pan Assay Interference Compounds (PAINS) From Screening Libraries and for Their Exclusion in Bioassays,” Journal of Medicinal Chemistry 53, no. 7 (2010): 2719–2740. 10.1021/jm901137j 20131845

[24] N. Brown , H. Zehender , K. Azzaoui , A. Schuffenhauer , L. M. Mayr , and E. Jacoby , “A Chemoinformatics Analysis of Hit Lists Obtained From High‐Throughput Affinity‐Selection Screening,” Journal of Biomolecular Screening 11, no. 2 (2006): 123–130. 10.1177/1087057105283579 16361695

[25] M. Snarey , N. K. Terrett , P. Willett , and D. J. Wilton , “Comparison of Algorithms for Dissimilarity‐Based Compound Selection,” Journal of Molecular Graphics and Modelling 15, no. 6 (1997): 372–385. 10.1016/s1093-3263(98)00008-4 9704300

[26] M. Congreve , R. Carr , C. Murray , and H. Jhoti , “A “Rule of Three” for Fragment‐Based Lead Discovery?” Drug Discovery Today 8, no. 19 (2003): 876–877. 10.1016/s1359-6446(03)02831-9 14554012

[27] P. Englert and P. Kovács , “Efficient Heuristics for Maximum Common Substructure Search,” Journal of Chemical Information and Modeling 55, no. 5 (2015): 941–955. 10.1021/acs.jcim.5b00036 25865959

[28] F. E. Maranzana , “On the Location of Supply Points to Minimize Transportation Costs,” IBM Systems Journal 2, no. 2 (1963): 129–135.

[29] F. N. Edfeldt , R. H. Folmer , and A. L. Breeze , “Fragment Screening to Predict Druggability (Ligandability) and Lead Discovery Success,” Drug Discovery Today 16, no. 7‐8 (2011): 284–287. 10.1016/j.drudis.2011.02.002 21315179

[30] R. Todeschini and V. Consonni , “Handbook of Molecular Descriptors. Methods and Principles in Medicinal Chemistry, Volume 11,” European Journal of Medicinal Chemistry 36, no. 11‐12 (2001): 966.

[31] H. Gohlke and G. Klebe , “Approaches to the Description and Prediction of the Binding Affinity of Small‐Molecule Ligands to Macromolecular Receptors,” Angewandte Chemie International Edition 41, no. 15 (2002): 2644–2676. 10.1002/1521-3773(20020802)41:15<2644::AID-ANIE2644>3.0.CO;2-O 12203463

[32] “RDKit: Open‐source cheminformatics,” https://www.rdkit.org/.

[33] S. A. Wildman and G. M. Crippen , “Prediction of Physicochemical Parameters by Atomic Contributions,” Journal of Chemical Information and Computer Sciences 39, no. 5 (1999): 868–873.

[34] D. E. Clark , “Rapid Calculation of Polar Molecular Surface Area and Its Application to the Prediction of Transport Phenomena. 1. Prediction of Intestinal Absorption,” Journal of Pharmaceutical Sciences 88, no. 8 (1999): 807–814. 10.1021/js9804011 10430547

[35] A. Vulpetti , G. Landrum , S. Rüdisser , P. Erbel , and C. Dalvit , “ ^19^F NMR Chemical Shift Prediction With Fluorine Fingerprint Descriptor,” Journal of Fluorine Chemistry 131, no. 5 (2010): 570–577.

[36] A. Razgulin and S. Mecozzi , “Binding Properties of Aromatic Carbon‐Bound Fluorine,” Journal of Medicinal Chemistry 49, no. 26 (2006): 7902–7906. 10.1021/jm0600702 17181174

[37] P. Zhou , J. Zou , F. Tian , and Z. Shang , “Fluorine Bonding ‐How Does it Work in Protein‐Ligand Interactions?” Journal of Chemical Information and Modeling 49, no. 10 (2009): 2344–2355. 10.1021/ci9002393 19788294

[38] Y. Tokunaga , K. Takeuchi , and I. Shimada , “Forbidden Coherence Transfer of ^19^F Nuclei to Quantitatively Measure the Dynamics of a ${\mathrm{CF}}_{3}$‐Containing Ligand in Receptor‐Bound States,” Molecules 22, no. 9 (2017): 1492. 10.3390/molecules22091492 PMC6151541 28880244

[39] Y. Mizukoshi , K. Takeuchi , M. Arutaki , et al., “Improvement of Ligand Affinity and Thermodynamic Properties by NMR‐Based Evaluation of Local Dynamics and Surface Complementarity in the Receptor‐Bound State,” Angewandte Chemie International Edition 55, no. 47 (2016): 14606–14609. 10.1002/anie.201607474 27762089

[40] W. Rut , K. Groborz , L. Zhang , et al., “SARS‐CoV‐2 M^pro^ Inhibitors and Activity‐Based Probes for Patient‐Sample Imaging,” Nature Chemical Biology 17, no. 2 (2021): 222–228. 10.1038/s41589-020-00689-z 33093684

[41] Y. Harrar , C. Bellini , and J.‐D. Faure , “FKBPs: at the Crossroads of Folding and Transduction,” Trends in Plant Science 6, no. 9 (2001): 426–431. 10.1016/s1360-1385(01)02044-1 11544132

[42] R. Leonardi , Y.‐M. Zhang , C. O. Rock , and S. Jackowski , “Coenzyme A: Back in Action,” Progress in Lipid Research 44, no. 2 (2005): 125–153. 10.1016/j.plipres.2005.04.001 15893380

[43] R. J. Moreau , C. K. Skepper , B. A. Appleton , et al., “Fragment‐Based Drug Discovery of Inhibitors of Phosphopantetheine Adenylyltransferase from Gram‐Negative Bacteria,” Journal of Medicinal Chemistry 61, no. 8 (2018): 3309–3324. 10.1021/acs.jmedchem.7b01691 29498517

[44] L. R. Thomas , C. M. Adams , J. Wang , et al., “Interaction of the Oncoprotein Transcription Factor MYC With Its Chromatin Cofactor WDR5 is Essential for Tumor Maintenance,” Proceedings of the National Academy of Sciences 116, no. 50 (2019): 25260–25268. 10.1073/pnas.1910391116 PMC6911241 31767764

[45] M. Dang and J. Song , “CTD of SARS‐CoV‐2 N Protein is a Cryptic Domain for Binding ATP and Nucleic Acid That Interplay in Modulating Phase Separation,” Protein Science 31, no. 2 (2022): 345–356. 10.1002/pro.4221 PMC8661809 34734665

[46] R. Zhou , R. Zeng , A. von Brunn , and J. Lei , “Structural Characterization of the C‐Terminal Domain of SARS‐CoV‐2 Nucleocapsid Protein,” Molecular Biomedicine 1, no. 1 (2020): 2. 10.1186/s43556-020-00001-4 PMC7406681 34765991

[47] A. Cléry , M. Krepl , C. Nguyen , et al., “Structure of SRSF1 RRM1 Bound to RNA Reveals an Unexpected Bimodal Mode of Interaction and Explains Its Involvement in SMN1 Exon7 Splicing,” Nature Communications 12, no. 1 (2021): 428. 10.1038/s41467-020-20481-w PMC7813835 33462199

[48] N. De Silva , T. Fargason , Z. Zhang , T. Wang , and J. Zhang , “Inter‐domain Flexibility of Human Ser/Arg‐Rich Splicing Factor 1 Allows Variable Spacer Length in Cognate RNA's Bipartite Motifs,” Biochemistry 61, no. 24 (2022): 2922–2932. 10.1021/acs.biochem.2c00565 36454680

[49] S. Das and A. Krainer , “Emerging functions of SRSF1, Splicing Factor and Oncoprotein, in RNA Metabolism and Cancer,” Molecular Cancer Research 12, no. 9 (2014): 1195–1204. 10.1158/1541-7786.MCR-14-0131 PMC4163531 24807918

[50] M. Congreve , C. J. Langmead , J. S. Mason , and F. H. Marshall , “Progress in Structure Based Drug Design for G Protein‐Coupled Receptors,” Journal of Medicinal Chemistry 54 (2011): 4283–4311. 10.1021/jm200371q PMC3308205 21615150

[51] T. Tengs , A. B. Kristoffersen , T. R. Bachvaroff , and C. M. Jonassen , “A Mobile Genetic Element With Unknown Function Found in Distantly Related Viruses,” Virology Journal 10, no. 1 (2013): 132. 10.1186/1743-422X-10-132 PMC3653767 23618040

[52] J. L. Childs‐Disney , X. Yang , Q. M. Gibaut , Y. Tong , R. T. Batey , and M. D. Disney , “Targeting RNA Structures With Small Molecules,” Nature Reviews Drug Discovery 21, no. 10 (2022): 736–762. 10.1038/s41573-022-00521-4 PMC9360655 35941229

[53] S. Sreeramulu , C. Richter , H. Berg , et al., “Exploring the Druggability of Conserved RNA Regulatory Elements in the SARS‐CoV‐2 Genome,” Angewandte Chemie International Edition 60, no. 35 (2021): 19191–19200. 10.1002/anie.202103693 PMC8426693 34161644

[54] A. Palmioli , P. Sperandeo , A. Polissi , and C. Airoldi , “Targeting Bacterial Biofilm: A New LecA Multivalent Ligand with Inhibitory Activity,” ChemBioChem 20, no. 23 (2019): 2911–2915. 10.1002/cbic.201900383 31216375

[55] E. Siebs , E. Shanina , S. Kuhaudomlarp , et al., “Targeting the Central Pocket of the Pseudomonas aeruginosa Lectin LecA,” ChemBioChem 23, no. 3 (2022): e202100563. 10.1002/cbic.202100563 PMC9300185 34788491

[56] N. S. Troelsen , E. Shanina , D. Gonzalez‐Romero , et al., “The 3F Library: Fluorinated ${\mathrm{Fsp}}^{3}$‐Rich Fragments for Expeditious NMR Based Screening,” Angewandte Chemie International Edition 59, no. 6 (2020): 2204–2210. 10.1002/anie.201913125 31724281

[57] M. Mayer and B. Meyer , “Group Epitope Mapping by Saturation Transfer Difference NMR to Identify Segments of a Ligand in Direct Contact With a Protein Receptor,” Journal of the American Chemical Society 123, no. 25 (2001): 6108–6117. 10.1021/ja0100120 11414845

[58] J. Yan , A. D. Kline , H. Mo , E. R. Zartler , and M. J. Shapiro , “Epitope Mapping of Ligand‐Receptor Interactions by Diffusion NMR,” Journal of the American Chemical Society 124, no. 34 (2002): 9984–9985. 10.1021/ja0264347 12188651

[59] C. Post , “Exchange‐Transferred NOE Spectroscopy and Bound Ligand Structure Determination,” Current Opinion in Structural Biology 13, no. 5 (2003): 581–588. 10.1016/j.sbi.2003.09.012 14568612

[60] J. H. Overbeck , W. Kremer , and R. Sprangers , “A Suite of Based Relaxation Dispersion Experiments to Assess Biomolecular Motions,” Journal of Biomolecular NMR 74, no. 12 (2020): 753–766. 10.1007/s10858-020-00348-4 PMC7701166 32997265

[61] D. González‐Ruiz and H. Gohlke , “Steering Protein‐Ligand Docking With Quantitative NMR Chemical Shift Perturbations,” Journal of Chemical Information and Modeling 49, no. 10 (2009): 2260–2271. 10.1021/ci900188r 19795907

