# Supplementary material for: A Comprehensive 19F NMR Framework for Fragment‐Based Drug Discovery: The Validated Screening Library OpenFL600 and Efficient Affinity Ranking by CSAR
Source: Angew Chem Int Ed Engl. 2026 Jul 23;65(39):e9964915. doi: 10.1002/anie.9964915 (PMC13592316; doi:10.1002/anie.9964915)
Supplement: Supplementary file 1 [file ANIE-65-e9964915-s001.pdf]

Supplementary information

A Comprehensive ^{19}F NMR Framework for Fragment-Based Drug Discovery: The Validated Screening Library OpenFL600 and Efficient Affinity Ranking by CSAR

Dr. Simon H. Rüdisser, Gabriela Stadler, Dr. Giacomo Padroni, Daria Barbash, Marco Ruckstuhl, Nils Lorz, Dr. Christina Jordan, Sarah Skibinski, Timo T. Stühlinger, Sarah E. Kratzwald, Katharina M. Siess, Dr. Sven Brüschweiler, Dr. Gerald Platzer, Dr. Antoine Cléry, Prof. Robert Konrat, Prof. Frédéric H-.T. Allain, Dr. Alvar D. Gossert

Simon H. Rüdisser
E-mail: simon.ruedisser@biol.ethz.ch
Alvar D. Gossert
E-mail: alvar.gossert@biol.ethz.ch
ETH Zürich, 8093 Zürich, Switzerland

1 List of associated files

Table S1 Description of files and computer programs which are provided in the supplementary information or at https://github.com/SimonRuedisser/19F_NMRscreening.

filename	software	description
OtavaEnamine_combine_MCSSC_CF_v2	KNIME 4.3.4	workflow for selecting CF fragments
OtavaEnamine_combine_MCSSC_CF3_v1	KNIME 4.3.4	workflow for selecting CF_3 fragments
FHit_analysis	KNIME 4.3.4	workflow for calculating molecular descriptor of hits
OpenFL600_CF3_mixtures.xlsx	MS Excel	CF_3 mixtures
OpenFL600_CF_mixtures.xlsx	MS Excel	CF mixtures
OpenFL600_CF3_QC.tar.gz	Bruker Topspin 3	CF_3: ^{1}H and ^{19}F NMR data for quality control
OpenFL600_CF_QC.tar.gz	Bruker Topspin 3	CF: ^{1}H and ^{19}F NMR data for quality control
OpenFL600_CF3_QC.xlsx	MS Excel	CF_3: solubility, purity, chemical shift, aggregation properties
OpenFL600_CF_QC.xlsx	MS Excel	CF: solubility, purity, chemical shift, aggregation properties
19F_NMRscreening_hit_analysis.ipynb	Jupyter notebook	^{19}F screening hit analysis
CSA_CF3.xlsx	MS Excel	CSA values for CF_3 molecules calculated by DFT methods
CSA_CF.xlsx	MS Excel	CSA values for CF molecules calculated by DFT methods

The MS-Excel files, OpenFL600_CF_QC.tar.gz and OpenFL600_CF_QC.xlsx contain the experiment numbers for the reference spectra, which were measured as single molecules. The reference spectra for the small molecules in Bruker Topspin format (OpenFL600_CF3_QC.tar.gz, OpenFL600_CF_QC.tar.gz) are provided at the ETH research collection at https://doi.org/10.3929/ethz-c-000791936. The CSA values for all evaluated molecules calculated by the machine-learning approach can be found in the supporting information of Rüdisser *et al.*[1].

2 OpenFL600 ^{19}F NMR library design

2.1 Compound selection

The workflow for the selection of screening fragments has been implemented in the data analytics platform KNIME[2]. Molecular properties have been computed using the RDKit[3] nodes which are available in KNIME.

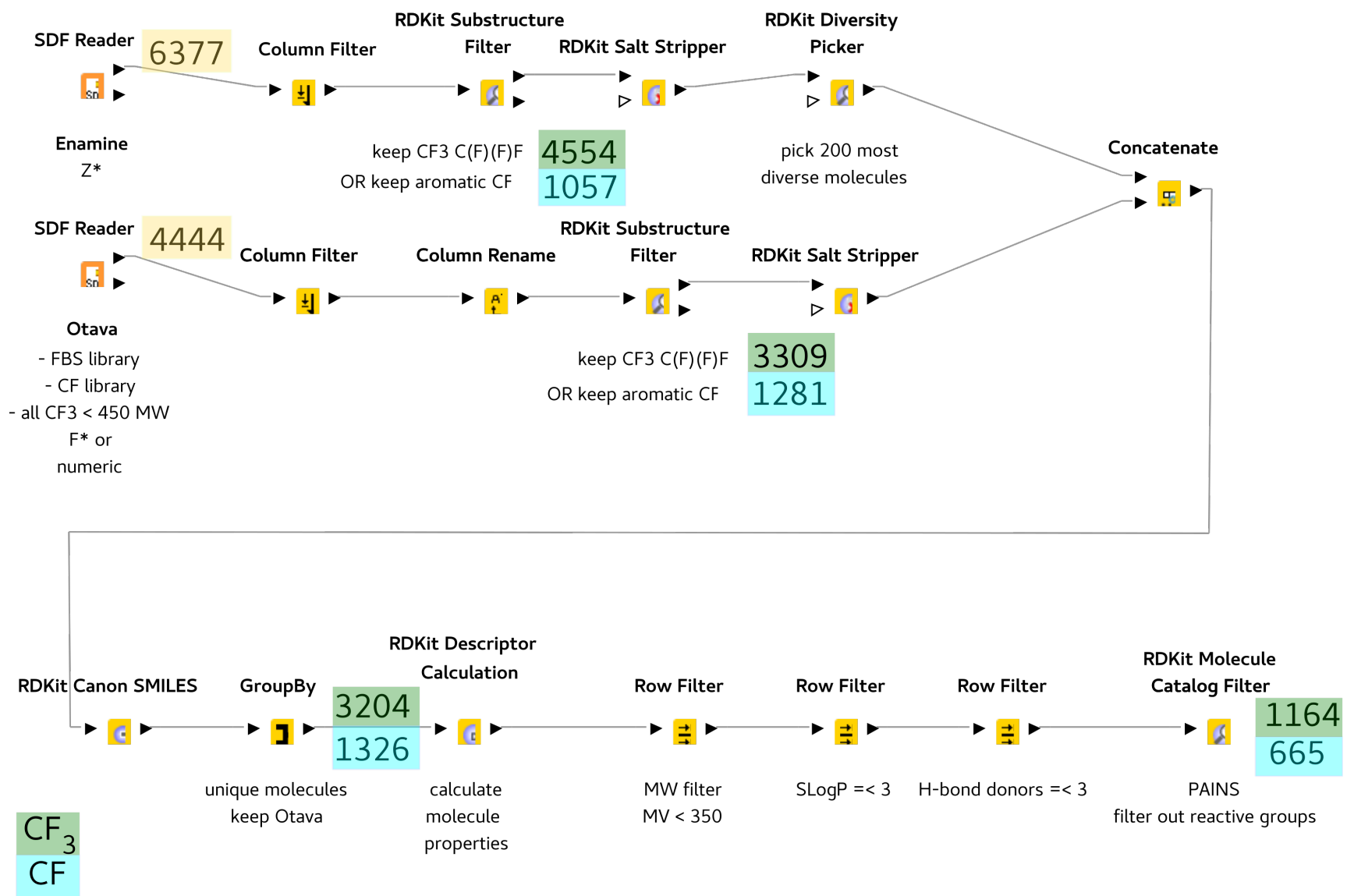

Fig. S1 The workflow for the selection of molecules for the CF and CF_3 library has been implemented in KNIME[2]. Molecules from two different vendors, Otava and Enamine, were combined to generate a diverse set of fragments. The majority of molecules has been selected from the Otava fluorine fragment screening collection and supplemented with the 200 most diverse molecules from the Enamine fluorine fragment screening collection. The RDkit[3] diversity picker has been used. The RDkit diversity picker selects diverse rows using the MaxMin algorithm[4] from an input table based on Tanimoto distances between fingerprints. Molecules with the potential to form a covalent bond with the target have been removed by applying the PAINS filter[5]. The number of molecules at each step of the workflow is indicated for the CF (cyan) and CF_3 (green) libraries.

2.2 Chemical structure, purity, solubility and aggregation properties determined by ^{1}H and ^{19}F NMR

The selected molecules, containing either CF and CF_3 groups, were characterized by ^{1}H NMR to assess solubility and purity, and by ^{19}F NMR to evaluate aggregation behavior. Compounds with adequate solubility, high purity, and a low propensity for aggregation were advanced into the screening library. Additionally, the measured ^{19}F chemical shifts were subsequently used to group molecules into mixtures that avoided signal overlap.

A 50 mM stock solution of each molecule was prepared in 90% DMSO-d_6 / 10% D_2O (v/v). For NMR analysis, a sample was made by diluting the stock solution into PBS buffer (pH 7.4, containing 10% D_2O and 100 µM DSS) to a final concentration of 1 mM. A 1D ^{1}H NMR spectrum was acquired using the

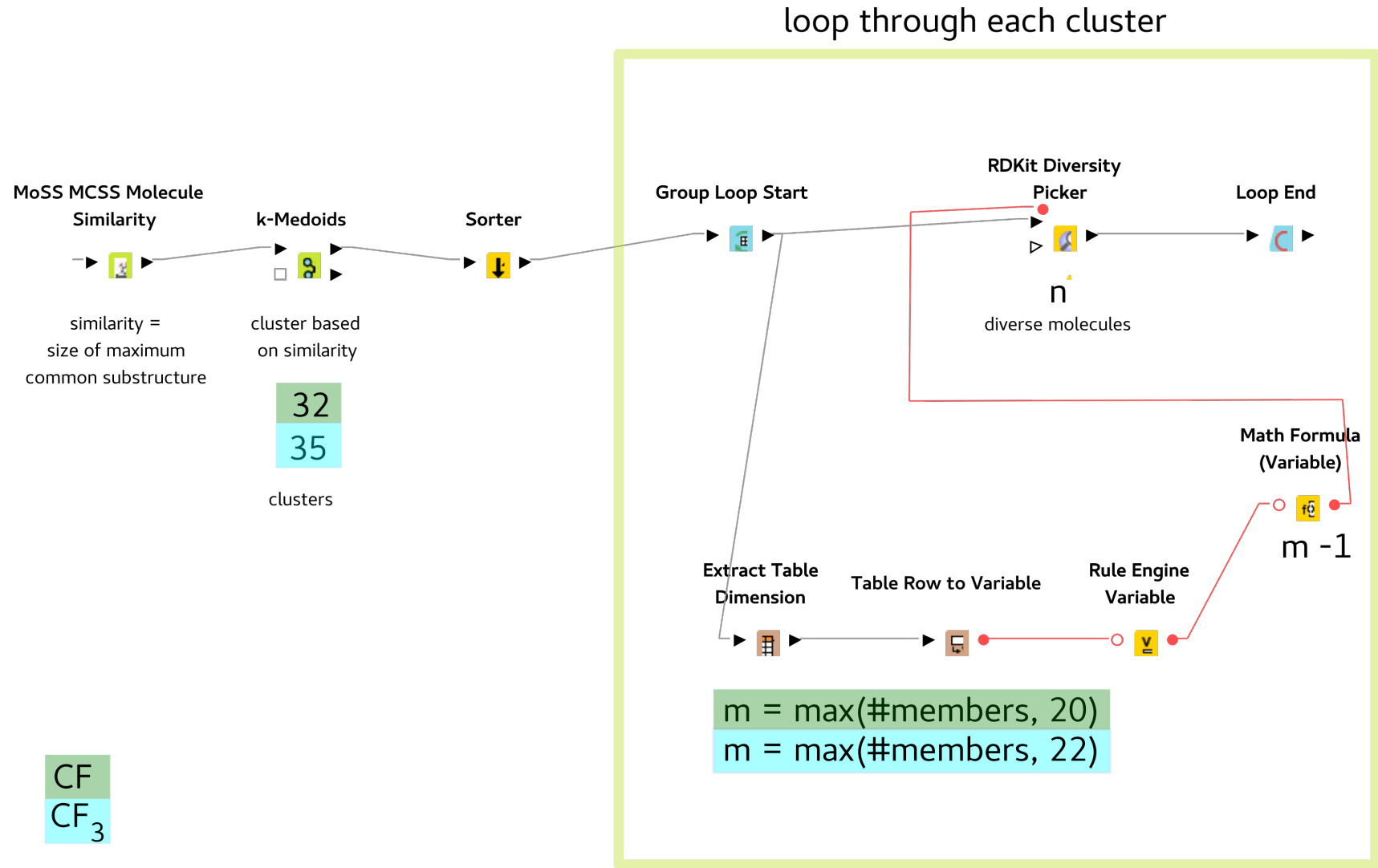

Fig. S2 Molecules have been grouped into clusters using the k-medoids method. The distance matrix for k-medoids clustering was computed from the MCSS (maximum common substructure) metric[6]. The number of clusters was set to 32 for CF and 35 for CF_3. For each of the obtained clusters at most 20 molecules for CF and 22 for CF_3 have been selected. If the number of cluster members was above the given upper limit, the most diverse subset for each cluster was selected using the RDkit diversity picker.

excitation sculpting water suppression method[7]. This spectrum was used to: (1) confirm consistency with the expected chemical structure, (2) calculate the molecule's concentration by referencing the DSS signal, and (3) determine the fraction of impurities present.

3 Screening targets

M^{pro}: The main protease (M^{pro}) of SARS-CoV-2 is an attractive target for antiviral drugs. The active site of this homodimeric cysteine protease includes the catalytic Cys145, which is targeted by covalent inhibitors [8]. Fragment screening campaigns have been reported for the discovery of both covalent and non-covalent inhibitors [9–11]. Screening our CF_3 library resulted in 10 hits which have been confirmed to bind to the protein target by
XL-ALSOFAST-[^{13}C,^{1}H]-HMQC[12] experiments using $^{13}C^{\epsilon}$ methionine labeled protein. Many of the hits contain at least one aromatic ring which potentially fits into one of the four sub-pockets of M^{pro}.

PPAT: Phosphopantetheine adenylyltransferase
(PPAT, CoaD) is a homohexameric protein with a molecular weight of 115.3 kDa for the hexamer. PPAT is an essential enzyme for coenzyme-A (CoA) synthesis that catalyzes the transformation of phosphopantetheine into dephospho-coenzyme A. In bacteria, CoA is a critical factor in the biogenesis of membrane lipids and is therefore a target for antibacterial drugs[13]. A recent study by Novartis shows how fragment-based drug discovery has been successfully applied for the discovery of PPAT inhibitors[14]. Notably, fragment 9 and 5 from Novartis, which bind to the panthetheine binding site, show a high similarity to a series of hits discovered in our research. In addition, we have discovered novel chemotypes, such as TFL318, that have not been previously described. The PPAT hits from our screening campaign show an above average of HBA, due to the presence of the heteroaromatic bicyclic rings.

SARS-CoV-2 N-CTD: The C-terminal domain (CTD) of the SARS-CoV-2 nucleocapsid (N) protein plays a crucial role in the virus's life cycle, including RNA binding and dimerization, which are essential for viral replication and assembly[15, 16]. The crystal structure of the SARS-CoV-2 N-CTD has been resolved to 2.0 Å, revealing an extensive interaction dimer in a domain-swapped manner, which is critical for its function[16]. This structural information is pivotal for understanding the biological functions of the N protein and for drug discovery efforts. Using NMR fragment-based drug discovery, 1,3-benzothiazol-2-amine derivatives were identified as potential antivirals. These compounds bind to the CTD, specifically to residues Arg 259, Trp 330, and Lys 338, which coincide with the nucleotide binding site, showing notable viral inhibition activity[17]. The fragment hit BNC-FL0304 which have been identified by screening the OpenFL600 library resembles a purine nucleotide and could therefore serve as a surrogate for guanine.

SRSF1-RRM12: SRSF1 (Serine/Arginine Splicing Factor 1), a member of the serine/arginine-rich (SR) protein family, plays a crucial role in RNA metabolism, including pre-mRNA splicing, mRNA stability, and translation[18–20]. It is involved in the regulation of over 1500 mRNA transcripts and is essential for the inclusion of specific exons in mature mRNA[21]. SRSF1 also has oncogenic properties, contributing to cancer progression by modulating alternative splicing of tumor suppressors and oncogenes[22, 23]. SRSF1 contains two RNA-recognition motifs (RRM1 and RRM2) and a C-terminal Arg/Ser-rich (RS) domain[18, 19]. The RRMs are crucial for RNA binding, with RRM2 playing a dominant role. The flexible linker between RRM1 and RRM2 allows for versatile RNA binding, accommodating various RNA sequences and structures[18, 19].

WDR5: WD repeat domain 5 (WDR5) is a member of the WD40-repeat protein family and represents a promising pharmacological target for diseases such as cancer and neurodegenerative disorders. The WIN site (WDR5 interaction motif[24]), which is responsible for chromatin recruitment, has been a major focus in drug discovery. Additionally, inhibition of MYC binding to the WBM site[25]—a binding site located opposite to the WIN site on the protein—offers a therapeutic opportunity for treating various cancers, including neuroblastoma, bladder cancer, and colorectal cancer. High-throughput screening[26] and fragment-based

screening by NMR[27] have been employed to identify ligands targeting the WBM site. In this study, we identified several ligands that induce chemical shift perturbations in a XL-ALSOFAST-[^{13}C,^{1}H]-HMQC[12]. This spectrum was acquired using a sample specifically labeled with ^{13}C on tyrosine, phenylalanine, tryptophan, leucine, and isoleucine residues. Ligand BNC-FL0052 shows chemical shift perturbations similar to those induced by OICR-9429 indicating binding in the MLL WIN motif-binding pocket[28].

β_1-adrenergic receptor: G protein-coupled receptors
(GPCRs) constitute a critical protein family involved in cell signaling in response to a diverse array of ligands. A significant number of small-molecule drugs targeting GPCRs have been developed across multiple therapeutic areas, and GPCR drug discovery remains a major focus in pharmaceutical research [29]. The GPCR β_1 adrenergic receptor (β_1AR) is predominantly expressed in cardiac tissue and plays a vital role in regulating blood pressure homeostasis. High-resolution structures of both partial antagonists and full agonists bound to β_1AR reveal interactions between the basic amine moiety and, in the case of agonists, the β-hydroxyl group, with residues Asp121 and Asn329. Additionally, all agonists form a hydrogen bond with Ser215, while numerous hydrophobic hot spots accommodate aromatic moieties (for a review, see ref [29]). NMR screening of our ^{19}F CF library resulted in an above average hit-rate. The hits show the characteristic features of β_1AR ligands, which include an aromatic moiety linked to a basic amine by an aliphatic linker. Two representative structures, BNC-FL0061 and BNC-FL0089, are shown in Figure **??**.

RNA: Targeting RNA is being actively pursued to expand the mode of action of small molecules. Binding pockets in RNA are distinct from those in proteins; for example, RNA has an enhanced electronegative surface potential and generally a smaller buried surface area compared to proteins[30]. Stem-loop 3 (SL3) of the U1 snRNA is known to interact with the RNA binding motif 39 (RBM39)[31]. RBM39 is overexpressed in several types of cancer and is essential for the survival of many cancer cells including Acute Myeloid Leukaemia (AML)[32]. The stem-loop II like motif (s2m) is part of the 3$'$ untranslated region of the SARS-Cov-2 viral genome[33].
Common chemotypes for RNA binders include 2-phenylindole, 2-phenylbenzimidazole, and 2-phenylimidazole. We refer to the works of Childs-Disney [34] and Sreeramulu [35], and the references therein. ^{19}F NMR screening of our library identified chemotypes similar to those described above: a heterocyclic aromatic system, such as triazolopyridine or quinazoline, linked by an aliphatic chain to a primary or secondary amine. The amine carries a positive charge at neutral pH and is presumed to interact with the negatively charged backbone oxygen.

FKBP12: FK506-binding proteins (FKBPs) constitute one of the three families of enzymes exhibiting peptidyl-prolyl *cis-trans* isomerase activity. Inhibitors of FKBP12 have demonstrated neuroprotective properties[36]. As a more recent example of fragment-based drug discovery utilizing protein-observed NMR, we direct readers to the work of Stebbins *et al.* [37]. In their study, the authors screened a fragment library comprising approximately 3000 molecules, identifying four hits with K_{D} values ranging from 15 to 167 μM. One of our ligands, BNC-FL0560 (Figure **??**), shows chemical similarity to the sulfonamide compound 4 in Figure 2[37]. BNC-FL0415 exhibits novel structural features not previously observed in other FKBP12 ligands.

LecA and LecB: LecA and LecB are carbohydrate-binding proteins (lectins) from *Pseudomonas aeruginosa* that play crucial roles in biofilm formation, which is a hallmark of chronic infections[38, 39]. LecA is a tetrameric and Ca^{2+}-dependent lectin that binds galactose. It has a unique helical structure that allows for specific binding interactions[38]. LecB is a Ca^{2+}-dependent lectin that binds fucose[40]. Efforts to develop inhibitors for LecA have focused on ligands which are based on carbohydrates due to their natural binding affinity. However, these ligands often have weak affinities, making drug development challenging. Tech-

niques like protein-observed ^{19}F NMR have been used to identify and improve the binding of low-affinity ligands[41]. To the best of our knowledge, only a limited number of non-carbohydrate ligands have been reported for either lectin. For LecA, one example is a class of catechol-based compounds, which act as promising carbohydrate mimetics[42]. More recently, Tolcapone and related molecules have been identified as a novel class of non-carbohydrate LecA ligands[43].

4 Protein and RNA production and purification

4.1 PPAT (CoaD)

Phosphopantetheine adenylyltransferase (PPAT or CoaD) was expressed with the sequence below, comprising the full length *E. coli* PPAT (UniProt ID: P0A6I6) with three additional N-terminal residues and eight additional C-terminal residues including an uncleavable His_6-tag. The amino acid sequence used for the expression of PPAT was designed to match the exact sequence used by Lingel *et al.*[44] for their ^{19}F NMR fragment screening. The protein was expressed using a pET-28b(+)-based plasmid purchased from BioCat GmbH, Heidelberg, Germany.

MDPMQKRAIYPGTFDPITNGHIDIVTRATQMFDHVILAIAASPSKKPMFTLEERVALAQQATAHLG
NVEVVGFSDLMANFARNQHATVLIRGLRAVADFEYEMQLAHMNRHLMPELESVFLMPSKEWSFIS
SSLVKEVARHQGDVTHFLPENVHQALMAKLAVDHHHHHH

E. coli BL21(DE3) Codon Plus RIL competent cells (Agilent) were transformed with plasmid DNA coding for PPAT and incubated overnight at 37°C on an agar plate containing kanamycin (50 mg/l). For the preculture, 50 ml of Luria Bertani (LB) medium containing chloramphenicol (34 mg/l) and kanamycin (50 mg/l) was inoculated with four colonies and incubated at 37°C for 20 h in a 500 ml baffled Erlenmeyer flask at 100 rpm shaking speed in an Infors HT Multitron shaking incubator. The optical density at 600 nm (OD 600) of the preculture was measured and 28 ml of the preculture was transferred to 950 ml of LB medium containing chloramphenicol and kanamycin in order to reach a start OD 600 of 0.1. The main culture was grown at 37°C in a 5 l baffled Erlenmeyer flask at 90 rpm shaking until OD 600 reached 0.8. The shaker was cooled to 18°C, and after an equilibration time of 30 min the culture was induced with 1 mM isopropyl-β-D-thiogalactopyranoside and 0.50 g of L-methionine-$^{13}C^{\epsilon}$ dissolved in 50 ml of LB containing chloramphenicol and kanamycin. The culture was grown at 18°C for 20 h at 90 rpm. Cells were harvested by centrifugation at 4’000 g for 15 min at 4°C (Beckmann Coulter Avanti JXN-26 centrifuge equipped with JLA8.1000 rotor) and stored at -80°C.

The protocol for PPAT purification was optimized based on a protocol from Proudfoot *et al.*[44]. The cell pellet was thawed on ice and resuspended in 25 ml lysis buffer (50 mM Tris pH 8.0, 300 mM NaCl, 5 mM imidazole, 10% glycerol, 1 mM DTT, one spatula tip of DNAse I, 1 mM PMSF). Cells were lysed with M-110L Microfluidizer (Microfluidics) in 4 cycles at 15’000 PSI. The obtained lysate was centrifuged at 27’000 g for 2 h at 4°C (Beckmann Coulter Avanti JXN-26 Centrifuge equipped with JLA25.50 rotor). The resulting supernatant was filtered through a 0.45 µm syringe filter and loaded onto a 5 ml Ni-NTA Superflow Cartridge (Qiagen) using ÄKTAxpress (GE Healthcare). The column was washed with binding buffer (50 mM Tris pH 8.0, 300 mM NaCl, 5 mM imidazole, 10% glycerol, 1 mM DTT) and eluted with a continuous gradient from binding buffer to elution buffer (50 mM Tris pH 8.0, 300 mM NaCl, 250 mM imidazole, 10% glycerol, 1 mM DTT). Purity and concentration of the protein were monitored by RP-HPLC using a continuous gradient from 80% buffer A (0.1% TFA in H_2O) and 20% buffer B (0.1% TFA in 90% MeCN, 10% H_2O) to 100% buffer B (Agilent 1100 Series equipped with POROS C8 column at 80°C and UV 215 detector). The protein was then dialyzed against dialysis buffer (20 mM sodium citrate pH 5.0, 1 mM DTT) to remove co-purified coenzyme A from the PPAT active site. For dialysis, the protein was first concentrated using two 10 kDa concentrators (Amicon Ultra-15, Merck Millipore) to a total volume of 5 ml ($\approx$ 550 µM). The protein was washed twice by dilution with 20 ml of dialysis buffer and subsequent concentration to 5 ml, and was then diluted with dialysis buffer to a total volume of 10 ml. The protein was dialyzed against 4 l of dialysis buffer using a 3.5 kDa MWCO dialysis tube (Spectra/Por 3 Standard RC, Spectrum Labs) for a total of 40 h, with the dialysis buffer being exchanged after 20 h. Following dialysis, the protein buffer was exchanged into NMR buffer (20 mM sodium phosphate pH 7.0, 1 mM DTT) using a HiPrep

26/10 Desalting column. Purity, concentration and yield of the protein were determined by RP-HPLC and NanoDrop ND-1000 Spectrophotometer (Thermo Scientific). Final monomeric concentration was adjusted to 100 µM. The protein was aliquoted, flash frozen in liquid nitrogen and stored at -80°C.

4.2 N-CTD

Expression and purification of N-CTD is described in Korn *et al.*[45].

4.3 LecA and LecB

Expression and Purification of LecA and LecB from Pseudomonas aeruginosa Plasmid DNA (pET-28b(+)), containing a His6-TEV-GB1-HRV3C-LecA(1–122) or His6-TEV-GB1-HRV3C-LecB(1–115) construct, was transformed into *E. coli* BL21(DE3) Codon Plus RIL cells (Agilent) by heat shock. LB agar plates, supplemented with chloramphenicol (34 µg/mL) and kanamycin (50 µg/mL), were used and isolation of colonies was carried out after incubation at 37°C over night. The precultures were prepared using 50 mL ^{15}N-enriched M9 minimal medium containing 1 g/L $^{15}NH_4Cl$, 34 µg/mL chloramphenicol and 50 µg/mL kanamycin and were inoculated with one colony, the culture was grown over night at 37°C in a shaker. This was followed by dilution of the preculture to 1 L ^{15}N-enriched M9 minimal medium to reach an OD600 of 0.2 and the cultures were grown to an OD600 of 0.6 while keeping at 37°C in a shaker. The temperature was reduced to 20°C and for induction of protein expression 1 mM isopropyl-β-D-thiogalactopyranoside was added after 30 min. Bacterial cells were harvested after 16 h by centrifugation for 10 min at 6000 × g and 4°C, and the cell pellet obtained was lysed in lysis buffer (50 mM Tris pH 8.0, 300 mM NaCl, 5 mM imidazole, 1 mM DTT, 1 mM PMSF and 10% (v/v) glycerol) supplemented with DNase I (AppliCHem) with an LM10 Microfluidizer (Microfluidics). The lysate was centrifuged for 45 min at 35,000 × g and 4°C and the supernatant was filtered with a 0.45 µm nitrocellulose membranes (Merck, MF-Millipore). The purification was performed by Ni-affinity chromatography at 4°C on a 5 mL Ni-NTA Superflow-Cartridge (Qiagen) preequilibrated with washing buffer (50 mM Tris pH 8.0, 300 mM NaCl, 5 mM imidazole, 1 mM DTT, 10% (v/v) glycerol). The elution buffer (50 mM Tris pH 8.0, 300 mM NaCl, 250 mM imidazole, 1 mM DTT and 10% (v/v) glycerol) containing the protein exchanged to the washing buffer on a HiPrep 26/10 Desalting Column (GE). For tag-cleavage human rhinovirus 3C protease (inhouse production) was added at a concentration of 50 µg/mL and the sample was incubated overnight at 4°C. Afterwards, protease and cleaved tag were removed by reverse Ni-affinity chromatography on a 5 mL Ni-NTA Superflow-Cartridge (Qiagen). The amount of protein solution was reduced to 800 µM using a 10 kDa Amicon-Ultra 4 mL centrifugal filter (Merck Millipore) and further purified by size exclusion chromatography (SEC) at 4°C on a Superdex 75 Increase 10/300 column (GE) with SEC buffer (50 mM Tris pH 7.4, 150 mM NaCl, 2 mM $CaCl_2$, 1 mM DTT). The retention volume of 11.2 mL was compatible with formation of a homo-tetramer for LecB. The LecA construct was designed to form a homodimer. Finally, the protein was concentrated to 230 µM in 10 kDa Amicon-Ultra 4 mL centrifugal filter (Merck Millipore), aliquoted, flash-frozen and stored at -80°C.

4.4 U1 snRNA SL3, s2m RNA

The RNA strands were synthesized by in vitro transcription from synthetic DNA templates containing the T7 promoter region. For s2m, the last two nucleotides at the 5' end of the template strand were modified to contain 2'OMe groups. The s2m DNA template was annealed with a shorter DNA strand containing the

T7 promoter region plus one G nucleotide. For U1 SL3, two fully complementary DNA strands containing the T7 promoter region were used instead. These synthetic DNAs were purchased from Microsynth, Switzerland. The in vitro transcription reactions (using in-house purified T7 polymerase) were optimized by varying DMSO, $MgCl_2$ and NTPs concentration. Optimized in vitro transcription reactions were incubated for at least 5 h at 37°C and supplemented with EDTA before anion exchange HPLC purification using a DNAPac-PA100 22 × 250 mm column heated to 85°C. After column equilibration and injection at 100% buffer A (6 M urea, 12.5 mM Tris–HCl, pH 7.4), a 0.25 min gradient to 15% buffer B (6 M urea, 12.5 mM Tris–HCl, pH 7.4, 0.5 M NaCl) was used to elute excess of mononucleotides. The RNA was eluted using a slower gradient from 15% to 73% buffer B over 12.5 min, and the column was washed to 100% buffer B and re-equilibrated to 100% buffer A before the following injection. The pure RNA fractions were identified by urea-PAGE, the RNAs isolated by butanol extraction and lyophilized before resuspension into the NMR buffer. The RNA constructs were annealed by heating to 98°C for 1 min and snap-cooled on ice for 10 min before use.

U1 SL3 template 1:
5′ GGC CAG TGA ATT CTA ATA CGA CTC ACT ATA GGG ATT TCC CCA AAT GTG GGA AAC TCC C

U1 SL3 template 2:
5′ GGG AGT TTC CCA CAT TTG GGG AAA TCC CTA TAG TGA GTC GTA TTA GAA TTC ACT GGC C

T7 promoter:
5′ TAA TAC GAC TCA CTA TAG

U1 SL3 RNA:
5′ GGG AUU UCC CCA AAU GUG GGA AAC UCC C

The stem-loop II like motif (s2m) was prepared using in-vitro translation as described in Padroni *et al.*[46].

s2m DNA template:
5′ 2′OMeGO2′MeGT TCA CTG TAC ACT CGA TCG TAC TCC GCG TGG CCT CGG TGA ACC TAT AGT GAG TCG TAT TA

s2m RNA:
5′ GGU UCA CCG AGG CCA CGC GGA GUA CGA UCG AGU GUA CAG UGA ACC

4.5 M^{pro}

SARS-CoV-2 main protease (M^{pro}) was expressed in *E. Coli* fully protonated with a uniform ^{15}N and L-methionine-$^{13}C^{\epsilon}$ labeling pattern. M^{pro} was expressed fused to an N-terminal Glutathione-S-transferase (GST)-tag and an uncleavable C-terminal His_6-tag as shown in the sequence below. The aim of the GST-tag was to increase protein yield by enhancing protein solubility during expression. The construct contained two M^{pro}-autocleavage sites and a Tobacco Etch Virus (TEV) protease cleavage site to remove the remaining GST-tag after expression. The bold fraction below indicates the sequence of the purified protein after final TEV cleavage. Compared to the wild-type sequence (blue, UniProt ID: P0DTD1[3264 - 3569]), the purified protein contained two additional residues at its N-terminus and eight additional residues including the His_6-tag at its C-terminus. The pET- 28a(+)-based plasmid used for M^{pro} expression was purchased from GenScript, New Jersey, United States[47].

MSPILGYWKIKGLVQPTRLLLEYLEEKYEEHLYERDEGDKWRNKKFELGLEFPNLPYYIDGDV
KLTQSMAIIRYIADKHNMLGGCPKERAEISMLEGAVLDIRYGVSRIAYSKDFETLKVDFLSKLP
EMLKMFEDRLCHKTYLNGDHVTHPDFMLYDALDVVLYMDPMCLDAFPKLVCFKKRIEAIP
QIDKYLKSSKYIAWPLQ↓GWQATFGGGDHPPKSDLEVLFQGPLGSAVLQ↓CSENLYFQ↓**GSS**
GFRKMAFPSGKVEGCMVQVTCGTTTLNGLWLDDVVYCPRHVICTSEDMLNPNYEDLLI
RKSNHNFLVQAGNVQLRVIGHSMQNCVLKLKVDTANPKTPKYKFVRIQPGQTFSVLAC
YNGSPSGVYQCAMRPNFTIKGSFLNGSCGSVGFNIDYDCVSFCYMHHMELPTGVHAGT
DLEGNFYGPFVDRQTAQAAGTDTTITVNVLAWLYAAVINGDRWFLNRFTTTLNDFNLV
AMKYNYEPLTQDHVDILGPLSAQTGIAVLDMCASLKELLQNGMNGRTILGSALLEDEFT
PFDVVRQCSGVTFQGPHHHHHH

Fig. S3 Color coded sequence. M^{pro} was expressed fused to an N-terminal Glutathione-S- transferase (GST)-tag (purple) and an uncleavable C-terminal His_6-tag (green). Autocleavage sites (pink primary and orange secondary cleavage site) were introduced as well as a Tobacco Etch Virus (TEV) protease cleavage site (red) to remove the remaining GST-tag after expression. The bold fraction indicates the sequence of the purified protein after final TEV cleavage.

The protocol for M^{pro} expression and purification was optimized based on a protocol provided by Dhiman Gosh[47]. *E. coli* BL21(DE3) Codon Plus RIL competent cells (Agilent) were transformed with plasmid DNA coding for M^{pro} and incubated overnight at 37°C on an agar plate containing kanamycin (50 mg/l). For preculture I, 2 ml of Luria Bertani (LB) medium containing chloramphenicol (34 mg/l) and kanamycin (50 mg/l) was inoculated with two colonies and incubated at 37°C for 4 h at 220 rpm shaking speed in a compact shaking incubator (Edmund Bühler KS 15 & TH 15). 1 l of M9 minimal medium containing chloramphenicol and kanamycin was prepared using 1 g of $^{15}NH_4Cl$ and 4 g of D-glucose as principal nitrogen and carbon sources, respectively. Preculture I was transferred into 50 ml of the M9 minimal medium (preculture II), which was incubated at 37°C overnight in a 500 ml baffled Erlenmeyer flask at 100 rpm in an Infors HT Multitron shaking incubator. For the main culture, preculture II was then transferred to 925 ml of M9 minimal medium and was grown at 37°C in a 5 l baffled Erlenmeyer flask at 90 rpm shaking until optical density at 600 nm (OD600) reached 0.7. The shaker was cooled to 18°C, and after an equilibration time of 30 min the culture was induced with 1 mM isopropyl-β-D-thiogalactopyranoside (IPTG) and 0.25 g of L-methionine-$^{13}C^{\epsilon}$ dissolved in 25 ml of M9 minimal medium were added. The culture was grown at 18°C for 20 h at 90 rpm. Cells were harvested by centrifugation at 4'000 g for 15 min at 4°C (Beckmann Coulter Avanti JXN-26 Centrifuge equipped with JLA8.1000 rotor) and stored at -80°C.

The cell pellet was thawed on ice and resuspended in 25 ml lysis buffer (50 mM Tris pH 8.0, 300 mM NaCl, 5 mM imidazole, 10% glycerol, 1 mM DTT, one spatula tip of DNAse I). Cells were lysed with M-110L Microfluidizer (Microfluidics) in 4 cycles at 15'000 PSI. The obtained lysate was centrifuged at 27'000 g for 2 h at 4°C (Beckmann Coulter Avanti JXN-26 centrifuge equipped with JLA25.50 rotor). The resulting supernatant was filtered through a 0.45 µm syringe filter and loaded onto a 5 ml Ni-NTA Superflow Cartridge (Qiagen) using ÄKTAxpress (GE Healthcare). The column was washed with binding buffer (50 mM Tris pH 8.0, 300 mM NaCl, 5 mM imidazole, 10% glycerol, 1 mM DTT) and eluted in a one step elution with elution buffer (50 mM Tris pH 8.0, 300 mM NaCl, 250 mM imidazole, 10% glycerol, 1 mM DTT). Buffer was exchanged back into binding buffer using a HiPrep 26/10 Desalting column. Purity and

concentration of the protein were monitored by RP-HPLC using a continuous gradient from 80% buffer A (0.1% TFA in H_2O) and 20% buffer B (0.1% TFA in 90% MeCN, 10% H_2O) to 100% buffer B (Agilent 1100 Series equipped with POROS C8 column at 80°C and UV 215 detector). TEV digestion was performed overnight at a 1 : 180 w/w ratio of TEV protease (His_6-tagged, lab-internal production) to M^{pro}. Protein concentration and cleavage were monitored by RP-HPLC. The protein was concentrated using a 10 kDa concentrator (Amicon Ultra-15, Merck Millipore) and was further purified by size exclusion chromatography (SEC) in SEC buffer (50 mM sodium phosphate pH 7.55, 50 mM NaCl) through a Superdex 75 16/600 column. 1 L SEC buffer was prepared with 10 ml 5 M NaCl, 42.75 ml 1 M Na_2HPO_4 and 7.25 ml 1 M NaH_2PO_4 according to Gomori[48]. Purity and correct molecular weight of the protein were monitored by RP-HPLC and SDS-PAGE using a precast Mini-PROTEAN TGX Stain-Free Gel (Bio-Rad) running electrophoresis at 220 V for 26 min in SDS buffer (25 mM Tris, 190 mM glycine, 3.5 mM SDS, pH 8.3). The protein was concentrated through a new 10 kDa concentrator, spiked with 1 mM DTT and diluted with 10% D_2O to a final monomeric concentration of 100 µM. Concentration and yield were determined by RP-HPLC and NanoDrop ND-1000 Spectrophotometer (Thermo Scientific). The protein was aliquoted, flash frozen in liquid nitrogen and stored at -80°C. After thawing, concentration of the protein was checked by NanoDrop and monodispersity by analytical SEC through a Superdex 200 Increase 10/300 column (using a Gilson HPLC system equipped with UV 280 detector).

4.6 β_1AR

β_1AR has been expressed and purified as described in Rößler *et al.*[49].

NMR experiments were performed in the presence of 0.01% Lauryl maltose neopentyl glycol (LMNG) and 0.001% Cholesteryl hemisuccinate (CHS) detergents.

4.7 WDR5

Truncated human WDR5 (residues 24-334), containing an N-terminal His6-tag followed by a tobacco etch virus (TEV) cleavage site, was expressed in *E. coli* BL21(DE3) in the presence of Kanamycin. For uniformly ^{15}N-labeled WDR5 cells were grown in M9 minimal media containing $^{15}NH_4Cl$ (1 g/L) as sole nitrogen source. For the uniformly ^{15}N and selective Leuδ-[$^{13}CH_3$], Ileδ1-[$^{13}CH_3$], Pheϵ-[^{13}CH], Tyrϵ-[^{13}CH] and Trpζ3-[^{13}CH] labeled WDR5 the M9 minimal media was supplemented with 1 g/L $^{15}NH_4Cl$, 100 mg/L 5-^{13}C-3,3,4,5,5,5-$^2H_6$-ketoisocaproate, 100 mg/L 4-^{13}C-3,3-$^2H_2$-α-ketobutyrat, 25 mg/L 3,5-$^{13}C_2$-2,4,6-$^2H_3$-L-phenylalanine, 25 mg/L 3,5-13C2-2,6-2H2-L-tyrosine and 20 mg/L 5-^{13}-4,6-$^2H_2$ anthranilic acid. Cells were grown at 37°C until an OD600 of approximately 0.8 was reached. The shaker temperature was then lowered to 18°C, and protein expression was induced with a final concentration of 0.2 mM IPTG for approximately 18 hours overnight. Cells were harvested and lysed by sonication in a lysis buffer containing 20 mM Tris-HCl (pH 7.5), 500 mM NaCl, 10 mM imidazole, 1 mM , 0.5% CHAPS, 1 mM PMSF, 5% glycerol, and Halt Protease Inhibitor Cocktail (Thermo Scientific). Lysate was subsequently clarified by centrifugation, filtered, and loaded onto a HisTrap FF crude (Cytiva) equilibrated in HisTrap Buffer A (20 mM Tris-HCl, 500 mM NaCl, 5% glycerol, pH 7.5). The protein was eluted with 250 mM imidazole. Protein-containing fractions were pooled, and the sample was buffer-exchanged into TEV-cleavage buffer (20 mM Tris-HCl, 150 mM NaCl, 5% glycerol, pH 7.5) via a HiPrep 10/60 Desalting column (Cytiva). The His6-tag was cleaved off overnight at 4°C using TEV protease. The His6-tag was removed by passing the sample through a second HisTrap FF crude (Cytiva) equilibrated in high-salt TEV-cleavage buffer (500 mM NaCl). The protein-containing flow-through was collected, concentrated to approximately 1 mL and injected onto a HiLoad 16/600 Superdex 75 pg column (Cytiva) equilibrated in 50 mM sodium phosphate (pH 6.5) and 50 mM NaCl.

4.8 FKBP12

2 mL of LB medium supplemented with 50 µg/mL kanamycin and 34 µg/mL chloramphenicol were inoculated using five colonies from a BL21 DE3 C+ RIL strain produced using a peT28b(+) carrying a His_6-Presc-FKBP12(1-108) construct (see below). After 3 hours of incubation at 37°C and 250 rpm the preculture was used to inoculate 240 mL of M9 medium (with 1 g/l $^{15}NH_4Cl$) which subsequently were incubated overnight at 37°C and 150 rpm. This second preculture was used to inoculate 3 times 1420 mL of M9 media, by adding 80 mL to each expression culture resulting in an initial OD600 of 0.12. The cultures were incubated at 37°C and 95 rpm until a OD600 of 0.61 was reached and the temperature was adjusted to 20°C. At an OD600 of 0.9, expression was induced by supplementation with 1 mM IPTG and carried on overnight (16 hours). The cultures were harvested by centrifugation at 5000 rpm and 4°C for 12 minutes using a JLA 8.100 rotor. Pellets were stored at -80°C until further processing.

MGSSHHHHHHLEVLFQGPGVQVETISPGDGRTFPKRGQTCVVHYTGMLEDGKKFDSSRDRNKPF
KFMLGKQEVIRGWEEGVAQMSVGQRAKLTISPDYAYGATGHPGIIPPHATLVFDVELLKLE

The pallets from an expression in *E. coli* were resuspended in an amount of buffer A (50 mM Tris-HCl pH 8.0, 300 mM NaCl, 1 mM DTT, 5 mM imidazole, 10% Glycerol) supplemented with 1 mM PMSF and DNase I that 0.2 g pallet were suspended in each mL. Lysis was done using four runs on a microfluidizer LM-10 at 100 MPa. The lysate was centrifuged for 45 minutes at 17'000 rpm using a JA 25.50 rotor. The supernatant was filtered through a 0.45 µm syringe filter. The filtrate was loaded on a Ni-NTA Superflow cartridge (CV = 5 mL), which was equilibrated with buffer A in advance. The column was washed with 8 CV buffer A and elution was done with buffer B (50 mM Tris-HCl pH 8.0, 300 mM NaCl, 1 mM DTT, 250 mM imidazole, 10% Glycerol). The eluate was subsequently desalted using a HiPrep 26/10 desalting column. The eluate was incubated with 1 µg/mL 3C-PreScission protease over night at 4°C. The cleaved sample was loaded on a reverse Ni-NTA Superflow cartridge and the flow-through as well as the wash were collected in fractions of 2 mL. The protein content of the fractions was double checked using an HPLC and the richest fractions were pooled (total of 30 mL). 1.9 mL of the pool were loaded onto a HiPrep Superdex75 16/60 which was previously equilibrated with 1.2 CV buffer (20 mM $NaPO_4$ pH 6.0, 100 mM NaCl, 1 mM DTT). Size exclusion was performed at 0.5 mL/minute and fractions were analysed using HPLC. The richest fractions were pooled and concentrated using an Amicon ultra 3k centrifugation filter.

4.9 GB1-SRSF1 RRM1+2

MQYKLILNGKTLKGETTTEAVDAATAEKVFKQYANDNGVDGEWTYDDATKTFTVTEGSHHHHH
HMENLYFQGGSMSGGGVIRGPAGNNDCRIYVGNLPPDIRTKDIEDVFYKYGAIRDIDLKNRRGGP
PFAFVEFEDPRDAEDAVYGRDGYDYDGYRLRVEFPRSGRGTGRGGGGGGGGGAPRGRYGPPSR
RSENRVVVSGLPPSGSWQDLKDHMREAGDVCYADVYRDGTGVVEFVRKEDMTYAVRKLDNTKF
RSHEGETAYIRVKVDG

SRSF1 RRM1+2 was fused to an N-terminal GB1 tag for increased solubility, followed by 6xHis tag for the purification. Recombinant protein was overexpressed in the BL21 (DE3) codon plus *E.coli* strain in either Luria Bertani (LB) supplemented with 50 µg/ml chloramphenicol, 50 µg/ml kanamycin. Bacteria were cultured at 37°C until reaching the O.D. of 0.6. Protein expression was induced by 1 mM IPTG, overnight at 22°C. During the growing and expression period, cell cultures were constantly shaken at 100 rpm (Multitron, HT Infors). Cell cultures were centrifuged (Avanti JXN-26, Beckman Coulter) at 4°C, 4 000 r.p.m for 10 min. Cell pellets were resuspended in 20 mL of the lysis buffer (NaPi 50 mM, NaCl 1 M, pH 8.0) per one liter of culture using a vortex, adding one tablet of protease inhibitor (Roche) per 40 mL of lysate. The lysate with 0.1 % of Triton x100 (Sigma Aldrich) was sonicated (HD 2070, Bandelin Sonoplus)

at 4°C, 3 times for 30 s, 85 % power, centrifuged at 17 000 r.p.m for 40 min (Avanti JXN-26, Beckman Coulter). Resulting supernatant was further purified on the Nickel affinity chromatography column (Ni-NTA Agarose, Qiagen) as following: equilibration of the column with 5 column volumes (cv) of lysis buffer or resuspension buffer, loading of supernatant, washing with 5 cv of 3 M NaCl, 5 cv of 20 mM Imidazole, 5 cv of buffer A (NaPi 20 mM, NaCl 800 mM, Imidazole 40 mM, L-Arginine 150 mM, L-Glutamate 150 mM, DTT 5 mM, pH 7.4), 3 cv of buffer B (NaPi 20 mM, NaCl 800 mM, Imidazole 500 mM, L-Arginine 150 mM, L-Glutamate 150 mM, DTT 5 mM, pH 7.4), 1 cv of 1 M Imidazole. GB1-SRSF1 RRM1+2 was eluted in buffer A fraction. Pure GB1 containing protein was dialyzed against the storage buffer (NaPi 20 mM, Urea 6 M, DTT 1 mM, pH 7.4) overnight at room temperature. Protein was concentrated with 10-kDa molecular mass cutoff Centricon devices (Vivascience Sartorius). Concentrations were calculated using the molar extinction coefficients (Expasy) and the absorbance measured at 280 nm (Nanodrop, ND-100, Spectrophotometer). Protein quality was verified on the 12 % SDS polyacrylamide gel.

5 NMR spectroscopy

5.1 NMR instrumentation

A probe with separate ^{19}F and ^{1}H channels (19F/1H) allows ^{1}H decoupling during ^{19}F acquisition, yielding a single line of high sensitivity. Therefore, measurements of the CF containing ligands were carried out on QCI-F Cryoprobes. The NMR experiments were performed on the following Bruker instruments: 600 MHz AVIIIHD equipped with a QCI 1H/19F-13C/15N-2H Cryoprobe (for 19F/1H double resonance experiments, *i.e.* decoupling), 500 MHz AVNEO equipped with a QCI 1H&19F-13C/15N/31P-2H Cryoprobe (tunable for ^{19}F or ^{1}H single resonance experiments) and 700 MHz AVNEO equipped with a QCI 1H/19F-13C/15N-2H Cryoprobe. Titrations and screening experiments were measured on the 600 MHz AVIIIHD spectrometer. All measurements were performed in NMR tubes with 3 mm diameter, at 298 K.

5.2 ^{19}F NMR relaxation experiments

Measurements of transverse relaxation rates were performed using simple $T_1\rho$-type experiments recorded as a pseudo2D in interleaved acquisition to avoid differential heating between scans with relaxation delays between 2.5 and 250 ms. Spin lock strengths between 8 and 10 kHz were applied in these experiments (ω_{SL} = 8 or 10 kHz). The pulse sequence is available in the supplementary material of Rüdisser *et al.*[50]. The experimental conditions for $T_1\rho$-type experiments for specific receptor molecules are provided in Table S2.

Table S2 Experimental conditions for relaxation rate measurements for CSAR.

receptor	c(receptor) / μM	c(ligand) / μM	T / K	buffer
M^{pro}	30	200	298.0	45 mM sodium phosphate pH 7.55, 45 mM NaCl
PPAT	5	200	298.0	18 mM sodium phosphate pH 7.0

All buffers contain 1 mM DTT (dithiothreitol, Merck ≥ 98 % D0632-10G), 10 % D_2O (Merck 99.8% 617385-1.107KG), 50 µM TFA (trifluor acetic acid, Acros Organics 99.5% 293811000 Lot: A0417100), 100 µM DSS-D_6 (sodium trimethylsilylpropanesulfonate, CIL 98 % DLM-8206-1).

5.3 ^{19}F NMR screening

The ^{19}F T_2 spin-echo screening experiments were performed using the following parameters.

For the CF library: The carrier frequency was set to -70 ppm with a sweep-width of 80 ppm. A total of 32,768 complex data points were acquired, and ^{1}H decoupling was applied using the WALTZ-16 sequence [51]. To ensure uniform excitation and refocusing across the broad ^{19}F spectral width, 90°and 180°BURBOP pulses [44] were employed. Experiments were acquired with a short (5 ms) and a long (200 ms) T_2 delay with 256 increments. The pre-scan delay was set to 1 s.

For the CF_3 library: The carrier frequency was set to -120 ppm with a sweep-width of 150 ppm, and 122,880 complex data points were acquired. To ensure uniform excitation and refocusing across the broad ^{19}F spectral width, 90°and 180°BURBOP pulses [44] were employed. Experiments were acquired with a short (5 ms) and a long (350 ms) T_2 delay with 256 increments. The pre-scan delay was set to 1 s.

Signal intensities of ^{19}F T_2 experiments with short and long T_2 delays were compared for the reference samples containing no receptor and samples containing receptor. A molecule is considered a hit if the intensity ration $[I_{long}/I_{short}(receptor)]/[I_{long}/I_{short}(reference)]$ is below 0.8.

5.4 ^{19}F NMR screening conditions

The ligand concentration is set according to the following considerations: the lower bound is determined by the signal to noise ratio of the instrument, and the upper bound is determined by the total DMSO concentration that is cumulatively added to a protein. The 40 μM for CF are due to the limit in DMSO, such that 24 compounds result in about 1.8 % DMSO. At this concentration an acceptable signal to noise ratio on the employed QCI-F Cryoprobe at 600 MHz (Bruker) is achieved. For CF_3, from the signal to noise ratio 15 μM might be sufficient, but here we prefer to have higher signal to noise, such that a concentration of 30 μM was chosen.
The receptor concentration was optimized according to the following considerations: a high receptor concentration leads to more hits due to an increased fraction of bound ligand. The molecular mass of the receptor also influences the number of hits. Higher molecular mass, slowly tumbling biomolecules result in a more pronounced increase in R_2 relaxation upon binding and therefore in increased hit rates. Receptor concentration is limited by the cost of producing the bio-molecule. Furthermore, unspecific binding is more likely to occur at high receptor concentration. Unspecific binders are undesirable and can be avoided by lowering the receptor concentration.
It is advisable to perform a pilot screen with a subset of the library using varying receptor concentrations. As a rule of thumb, we propose screening at receptor concentrations between 2 and 10 μM. For high-molecular-weight complexes, a concentration of 2 μM is typically sufficient to identify binders. For small proteins or RNA, concentrations of around 10 μM are typically required to detect binding. Table S3 shows the screening conditions for the various targets investigated in this study.

Table S3 Screening conditions for the OpenFL600 library

receptor	c(rec.) μM	c(CF) μM	c(CF3) μM	competitor	c(comp.) μM	pH	buffer	
MPRO	6	40				7.00	18 mM NaP	90 mM NaCl, 1 mM DTT
MPRO	6		30			7.00	18 mM NaP	90 mM NaCl, 1 mM DTT
PPAT	10	40				7.00	18 mM NaP	1 mM DTT
PPAT	10		30			7.00	18 mM NaP	1 mM DTT
N-CTD	10		40			7.50	25 mM Tris	200 mM NaCl, 1 mM , 5 mM $MgCl_2$
SRSF1	13	40		UCAUUGGAU	15	7.45	20 mM NaP	1 mM DTT, 1 M urea
SRSF1	10		30	UCAUUGGAU	13	7.45	20 mM NaP	1 mM DTT, 1 M urea
WDR5	10	40				6.53	50 mM NaP	50 mM NaCl
WDR5	10		30			6.53	50 mM NaP	50 mM NaCl
β_1-AR	2	40		pindolol	5	7.50	20 mM HEPES	300 mM NaCl, 0.01% LMNG, 0.001% CHS
FKBP12	6	40				6.00	20 mM NaP	100 mM NaCl, 1 mM DTT
FKBP12	6		30			6.00	20 mM NaP	100 mM NaCl, 1 mM DTT
U1a SL3	15		30			6.80	50 mM NaP	100 mM NaCl
s2mRNA	10		40			6.00	25 mM NaP	50 mM NaCl
LecA	10	40		β-phenyl-galactopyranose	30	7.50	50 mM Tris	150 mM NaCl, 1 mM DTT, 2 mM $CaCl_2$
LecA	10		30	β-phenyl-galactopyranose	30	7.50	50 mM Tris	150 mM NaCl, 1 mM DTT, 2 mM $CaCl_2$
LecB	10	40		fucose	30	7.50	50 mM Tris	150 mM NaCl, 1 mM DTT, 2 mM $CaCl_2$
LecB	10		30	fucose	30	7.50	50 mM Tris	150 mM NaCl, 1 mM DTT, 2 mM $CaCl_2$

NaP ... sodium phosphate, LMNG ... Lauryl Maltose Neopentyl Glycol, CHS ... 3β-Hydroxy-5-cholesten-3-hemisuccinat,
DTT ... dithiothreitol, TCEP ... tris(2-carboxyethyl)phosphin

5.5 NMR data processing

Data were processed using TopSpin 4.0.7. Peak picking of spectra was done with the nmrglue module for Python3 (Helmus and Jaroniec 2013, https://www.nmrglue.com). Fitting of relaxation data was performed with self-written Python3 scripts (www.python.org) using the scipy (www.scipy.org), numpy (www.numpy.org) and pandas (www.pandas.pydata.org) modules. The experimental conditions for specific receptor molecules are provided in Table S2.

5.6 Hit validation by target observation experiments

In this section, protein or RNA observation NMR data for hit validation are shown for PPAT (CoaD), M^{pro}, WDR5, FKBP12, s2m 3′-UTR.

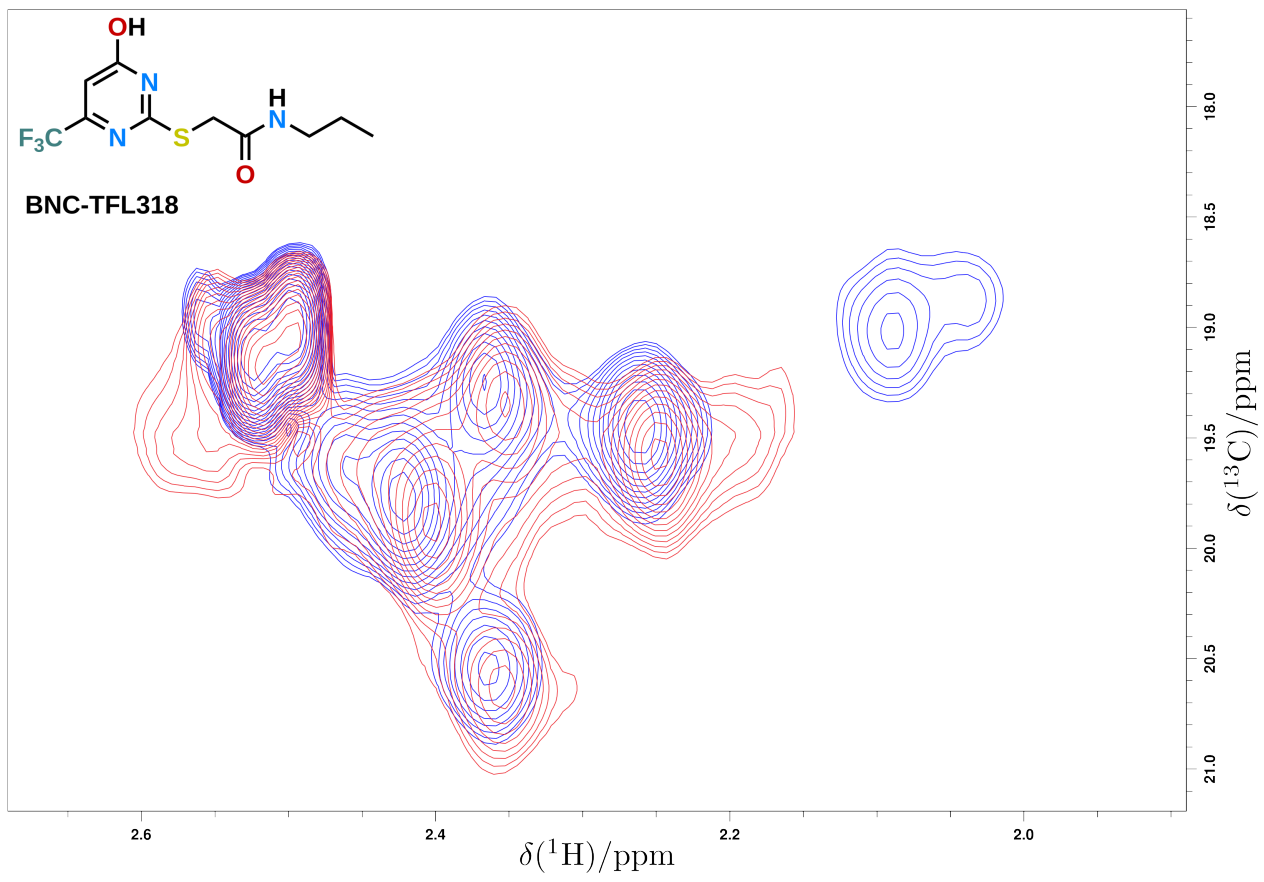

Fig. S4 Overlay of methyl regions from XL-ALSOFAST-[^{13}C,^{1}H]-HMQC spectra of 50 μM ^{13}C methyl-Met labeled PPAT for validation of hits from screening. Red: reference spectrum without addition of compound. Blue: spectra after addition of selected hits from the CF_3 library screening (TFL318 0.8 mM). The final buffer composition of all samples was 18 mM sodium phosphate pH 7.0, 1 mM DTT, 10% D_2O, 1.8% DMSO-D_6. Spectra were recorded at 308 K with 64 scans in 1.5 h on a 600 MHz Avance IIIHD spectrometer. The overlayed spectra show changes in the chemical shifts of different methyl groups in presence of all compounds, thereby validating the binding to PPAT.

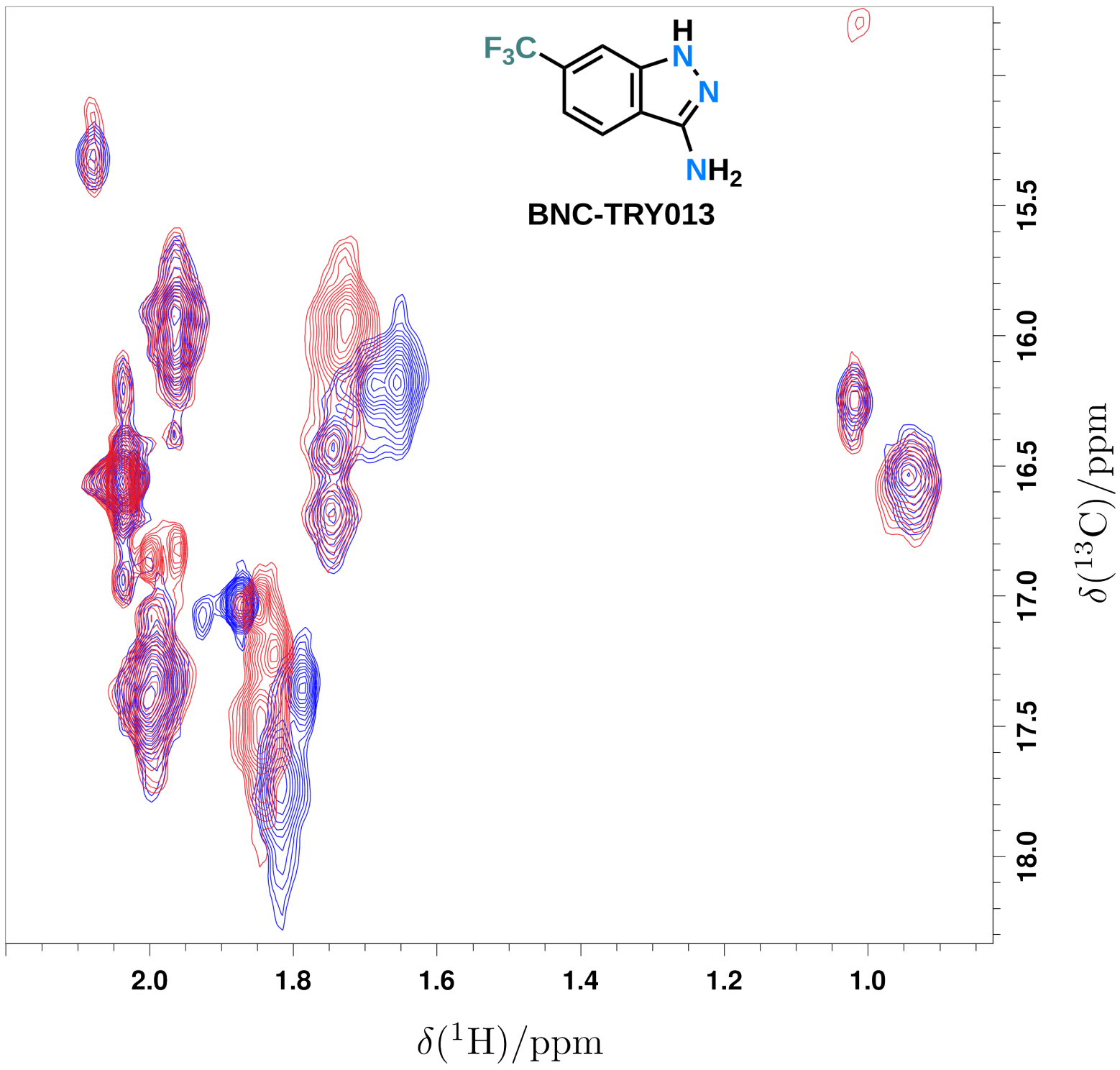

Fig. S5 Overlay of methyl regions from XL-ALSOFAST-[^{13}C,^{1}H]-HMQC spectra of u-^{15}N, ^{13}Cmethyl-Met labeled M^{pro} for validation of hits from screening. Red: reference spectra without addition of compound. Blue: spectra after addition of 5 mM of TRY013, a hit from the CF_3 library screening. The final buffer composition of all samples was 45 mM sodium phosphate, 45 mM NaCl, 1 mM DTT, 10% D2O, 1.8% DMSO-D6. Spectra were recorded at 92 μM M^{pro} concentration on a 700 MHz Avance IIIHD spectrometer. All spectra were measured at 298 K with 32 scans in 1.5 h.

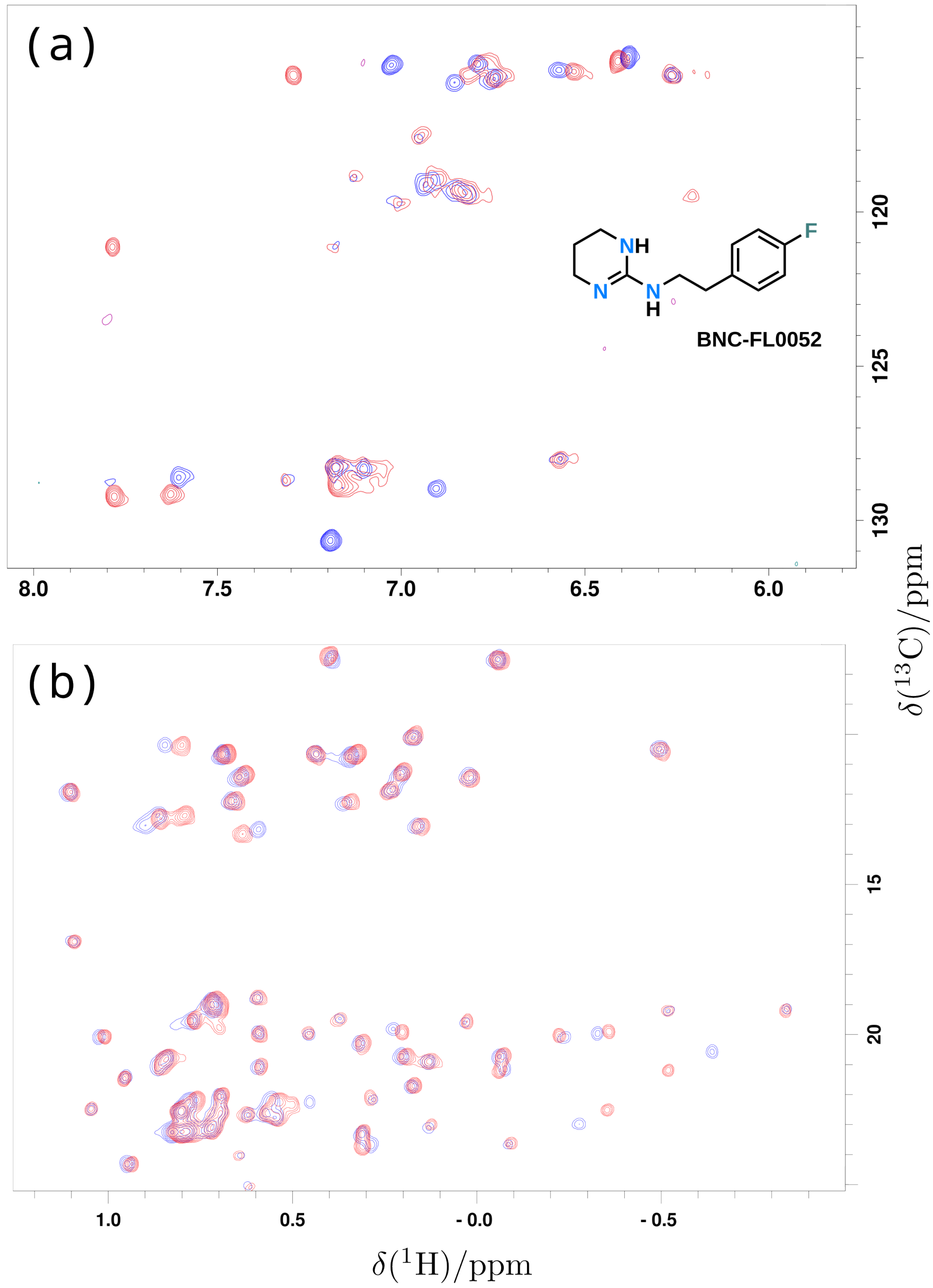

Fig. S6 XL-ALSOFAST-[^{13}C,^{1}H]-HMQC spectra overlay of the aromatic (a) and aliphatic (b) region of selective Leuδ-[$^{13}CH_3$], Ileδ1-[$^{13}CH_3$], Pheϵ-[^{13}CH], Tyrϵ-[^{13}CH] and Trpζ3-[^{13}CH] labeled WDR5 with the hit compounds BNC-FL0052 (blue) versus WDR5 with DMSO (reference, red). The spectra were recorded at 600 MHz at 298 K in 50 mM sodium phosphate buffer at pH 6.5,with 30 μM WDR5 and 1 mM ligand.

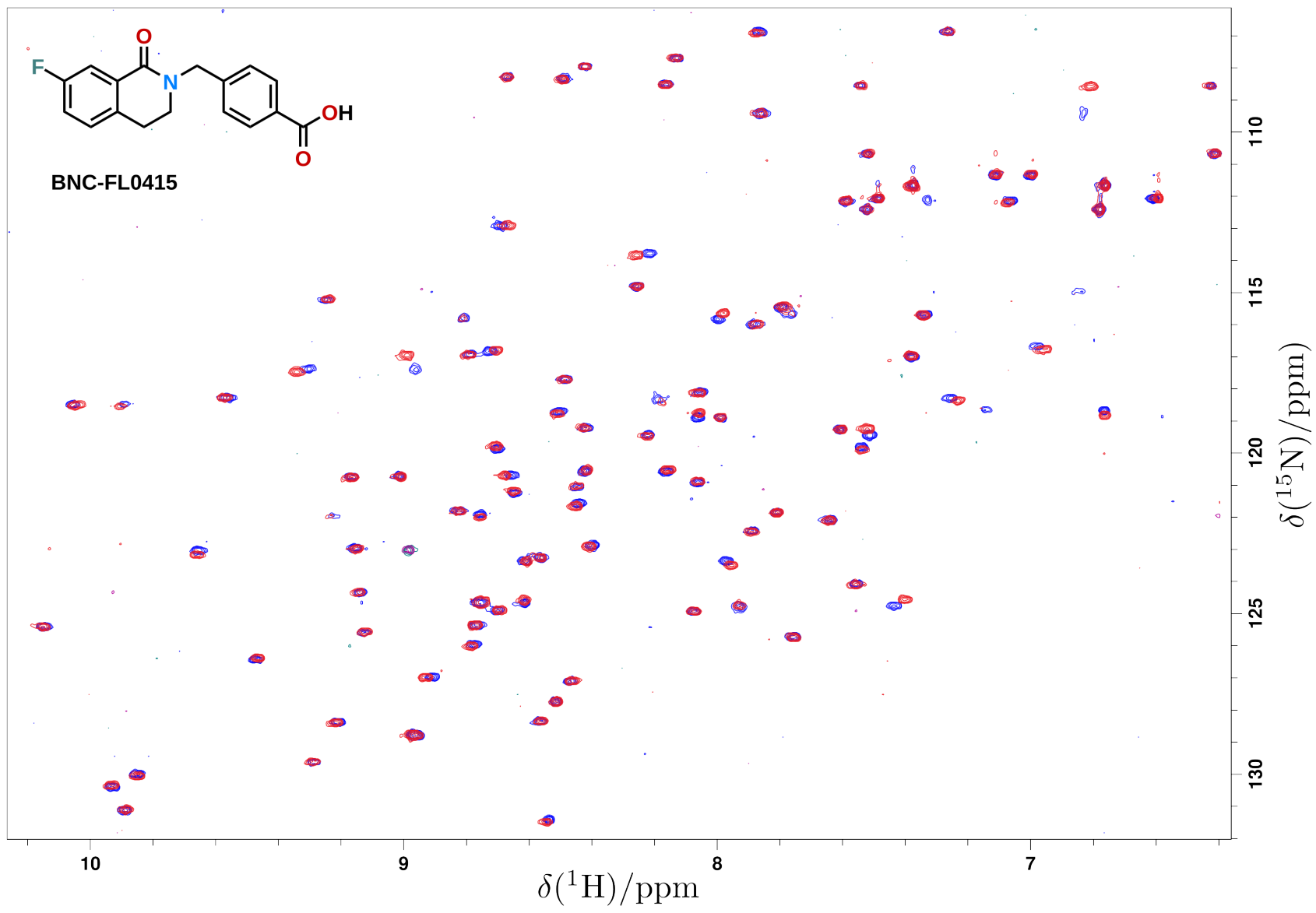

Fig. S7 2D [^{1}H, ^{15}N]-HSQC spectral overlay of FKBP with the hit compounds BNC-FL0415 (blue) versus FKBP with DMSO (reference, red). The spectra were recorded at 600 MHz at 298 K in 20 mM sodium phosphate buffer at pH 7.2, 1 mM with 80 μM FKBP and 1.1 mM ligand.

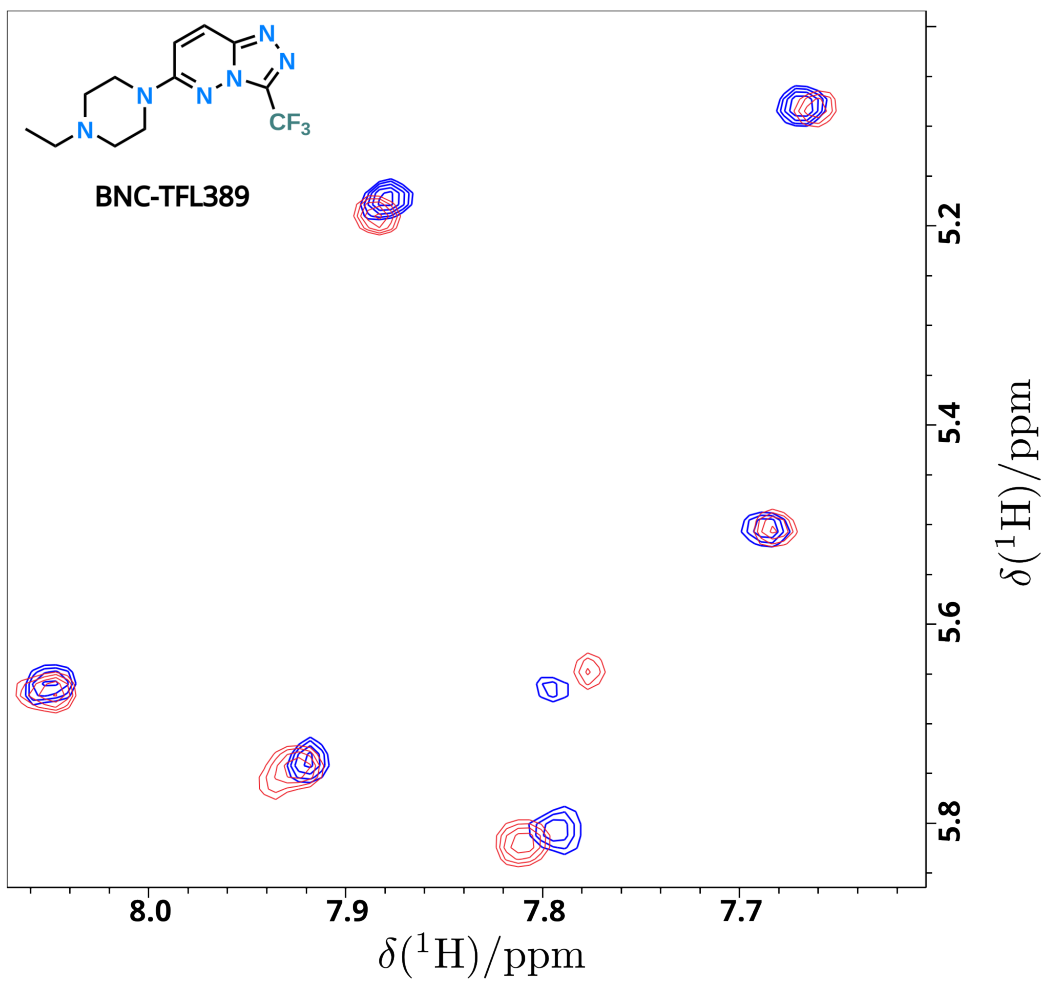

Fig. S8 2D [^{1}H,^{1}H]-TOCSY spectral overlay of s2m RNA with the hit compounds BNC-TFL389 (blue) versus s2m RNA with DMSO (reference, red). The spectra were recorded at 600 MHz at 298 K in 25 mM sodium phosphate pH 6.0, 50 mM NaCl with 50 μM RNA and 1 mM ligand. The spectrum shows the H5-H6 correlations for the pyrimidines cytidine and uridine.

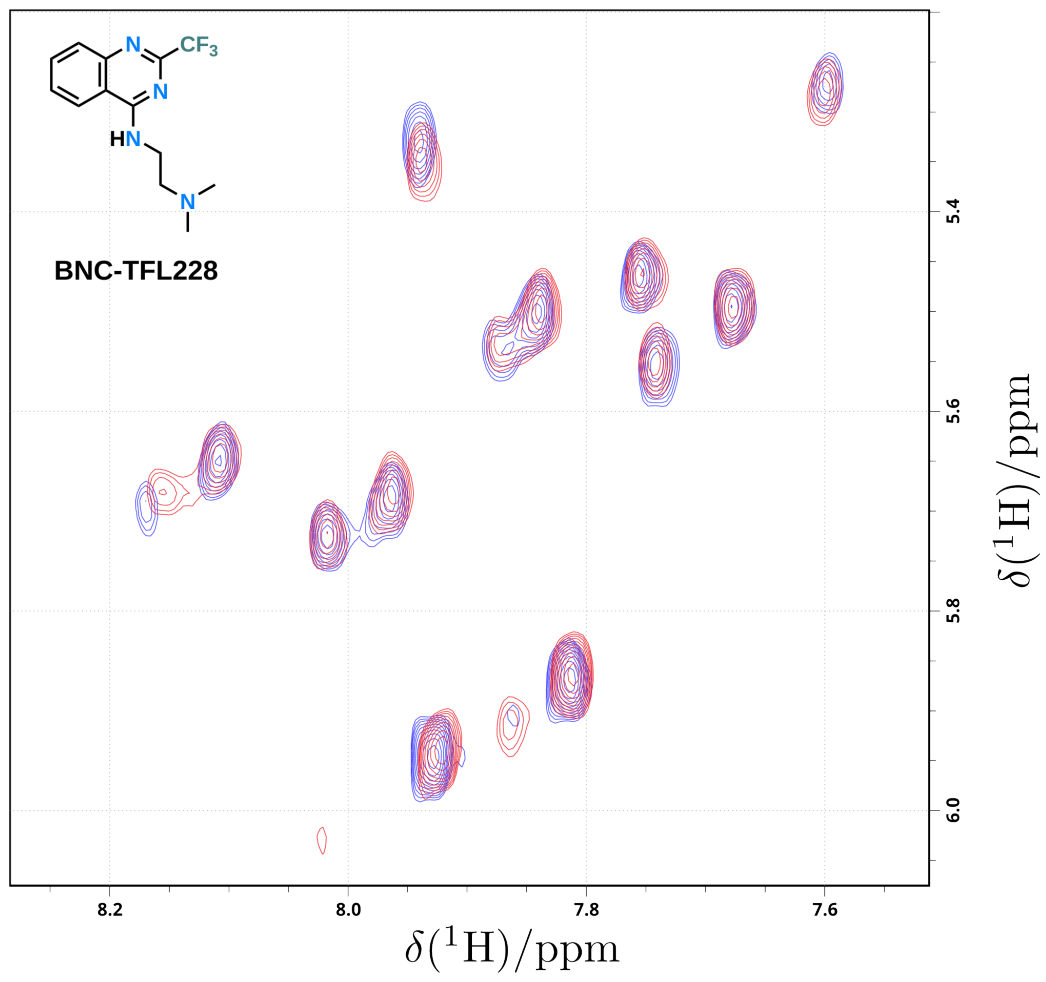

Fig. S9 2D [^{1}H,^{1}H]-TOCSY spectral overlay of SL3 RNA with the hit compounds BNC-BNC228 (blue) versus SL3 RNA with DMSO (reference, red). The spectra were recorded at 600 MHz at 298 K in 50 mM sodium phosphate pH 6.8, 100 mM NaCl with 25 μM RNA and 0.5 mM ligand. The spectrum shows the H5-H6 correlations for the pyrimidines cytidine and uridine.

6 ^{19}F CSA calculations for screening library molecules

6.1 ^{19}F CSA calculation with DFT methods

The three-dimensional structures of the small molecules have been generated from SMILES using openbable (www.openbable.org). Energy minimization was performed using the minimax minimizer (Witnotp)[52] and the Tripos all atom force field (TAFF)[53].

DFT calculations were performed using Gaussian09[54], following a protocol similar to that described by Rüdisser *et al.*[55]. To enhance computational efficiency, several modifications to the original protocol were made. Geometry optimizations were conducted at the M06L/def2TZVPP level of theory[56, 57], utilizing density fitting and an SMD implicit solvation model[58] to account for the dielectric effects of the surrounding water.

Chemical shielding tensors were computed at the energy minima using the Gauge-Independent Atomic Orbital (GIAO) method [59, 60], employing the M06 hybrid functional. The cc-pVTZ basis set was used for carbon (C), hydrogen (H), nitrogen (N), and oxygen (O), while the AUG-cc-pVTZ basis set was used for fluorine (F). For CF_3 groups, individual tensors for the fluorine atoms were averaged by static tensor averaging, as described in the supplementary information by Rüdisser *et al.*[55]. To compute the eigenvalues for the CSA, σ_{ii}, where $ii \in \{xx, yy, zz\}$ for CF_3 group, the components of the three tensors, σ_{ij} with $i, j \in \{x, y, z\}$, were averaged, and the eigenvalues of the resulting tensor were calculated. These computations were carried out using a self-written Python 3 script (www.python.org) utilizing the numpy module (www.numpy.org).

The CSA tensors have been computed for 187 CF_3 and for 324 CF fragments from the screening library using the EULER cluster computing infrastructure at the ETH Zürich (https://scicomp.ethz.ch/wiki/Euler). These results are used as training and test sets for random forest regression based on fluorine fingerprint descriptors.

7 CSAR affinity ranking based on R_2^{SL} measurements at two B_0 fields

The CSA contribution ($R_{2,\mathrm{CSA}}$) to transverse relaxation scales with the square of the magnetic field strength, whereas the DD contribution ($R_{2,\mathrm{DD}}$) is, to a first approximation, field-independent. Subtracting the relaxation rate at the first field, $R^{SL}_{2,B_{0_1}}$, from that at the second, $R^{SL}_{2,B_{0_2}}$, causes the dipolar contributions to cancel, thereby isolating the CSA effect[50] (Equation S1).

$$\begin{aligned} p_{\mathrm{b,app}} &= \frac{\Delta R_2^{\mathrm{SL}}}{\Delta R_{2,\mathrm{CSA,b}}} \\ &= \frac{\Delta R^{\mathrm{SL}}_{2,\mathrm{obs}}}{\Delta R_{2,\mathrm{CSA,b,calc}}} \end{aligned} \tag{S1}$$

Table S4 Experimental $\Delta R^{\mathrm{SL}}_{2,\mathrm{f,\ obs}}$ values are obtained from measurements at two different B_0 fields (11.75 T and 16.4 T). The theoretical $\Delta R_{2,\mathrm{calc}}$ relaxation rates are calculated based on a model for isotropic overall tumbling of the receptor[50]. Δ_σ and η values were obtained from DFT calculations. Note that all ligands contain a CF_3 group with $\eta = 0$.

receptor	ligand	Δ_σ, η / ppm	$\Delta R^{\mathrm{SL}}_{2,\mathrm{obs}}$ / s^{-1}	$\Delta R_{2,\mathrm{calc}}$ / s^{-1}	$p_{\mathrm{b,app}}$ / %
PPAT	TFL296	64.37, 0	1.57	176	0.89
PPAT	TFL328	66.69, 0	2.88	189	1.53
PPAT	TFL355	64.17, 0	1.49	175	0.85
M^{pro}	TFL071	54.55, 0	1.01	76.5	1.32
M^{pro}	TFL176	99.66, 0	0.601	255	0.24
M^{pro}	TFL331	62.31, 0	1.48	99.9	1.48
M^{pro}	TRY013	54.18, 0	0.761	75.5	1.01

The expected error in $p_{\mathrm{b,app}}$ is estimated based on the standard error of the relaxation rate measurements. This error is derived from the fitting of signal intensities across various relaxation delays and is dependent on the signal-to-noise ratio. Since all molecules were measured at the same concentration for a given receptor, the signal-to-noise ratio and therefore the fitting error remains approximately constant across the dataset, typically ranging from 1% to 2%. For the difference in rates, $\Delta R^{\mathrm{SL}}_{2,\mathrm{f,\ obs}}$, measured at two different magnetic fields, the total expected error is approximately 0.04 s^{-1}. At a typical difference in rates of 1 s^{-1} or more, this absolute error corresponds to a relative error of less than 4%. The expected error in the theoretical rate, $\Delta R_{2,\mathrm{calc}}$, is discussed in great detail in Rüdisser *et al.*[1]. As it remains approximately constant for a given receptor, it can be neglected for the purpose of affinity ranking. The error in the measured relaxation rates therefore translates into a relative error of 0.04 for $p_{\mathrm{b,app}}$ given in %.

References

(1) Rüdisser, S. H.; Stadler, G.; Gossert, A. D. *Angewandte Chemie International Edition* **2026**, DOI: 10.1002/anie.6036832.
(2) Berthold, M. R.; Cebron, N.; Dill, F.; Gabriel, T. R.; Kötter, T.; Meinl, T.; Ohl, P.; Thiel, K.; Wiswedel, B. *ACM SIGKDD Explorations Newsletter* **2009**, *11*, 26–31, DOI: 10.1145/1656274.1656280.
(3) RDKit: Open-source cheminformatics. https://www.rdkit.org/.
(4) Ashton, M.; Barnard, J.; Casset, F.; Charlton, M.; Downs, G.; Gorse, D.; Holliday, J.; Lahana, R.; Willett, P. *Quantitative Structure-Activity Relationships* **2002**, *21*, 598–604, DOI: 10.1002/qsar.200290002.
(5) Baell, J. B.; Holloway, G. A. *Journal of Medicinal Chemistry* **2010**, *53*, 2719–2740, DOI: 10.1021/jm901137j.
(6) Englert, P.; Kovács, P. *Journal of Chemical Information and Modeling* **2015**, *55*, 941–955, DOI: 10.1021/acs.jcim.5b00036.
(7) Hwang, T. L.; Shaka, A. J. *Journal of Magnetic Resonance, Series A* **1995**, *112*, 275–279, DOI: https://doi.org/10.1006/jmra.1995.1047.
(8) Rut, W.; Groborz, K.; Zhang, L.; Sun, X.; Zmudzinski, M.; Pawlik, B.; Wang, X.; Jochmans, D.; Neyts, J.; Młynarski, W.; Hilgenfeld, R.; Drag, M. *Nature Chemical Biology* **2021**, *17*, 222–228, DOI: 10.1038/s41589-020-00689-z.
(9) Douangamath, A. et al. *Nature Communications* **2020**, *11*, 1–11, DOI: 10.1038/s41467-020-18709-w.
(10) Cantrelle, F. X.; Boll, E.; Brier, L.; Moschidi, D.; Belouzard, S.; Landry, V.; Leroux, F.; Dewitte, F.; Landrieu, I.; Dubuisson, J.; Deprez, B.; Charton, J.; Hanoulle, X. *Angewandte Chemie - International Edition* **2021**, *60*, 25428–25435, DOI: 10.1002/anie.202109965.
(11) Altincekic, N.; Jores, N.; Löhr, F.; Richter, C.; Ehrhardt, C.; Blommers, M.; Berg, H.; Öztürk, S.; Gande, S.; Linhard, V.; Sreeramulu, S.; Schwalbe, H. *ACS Chemical Biology* **2024**, *19*, 563–574, DOI: 10.1021/acschembio.3c00720.
(12) Rößler, P.; Mathieu, D.; Gossert, A. D. *Angewandte Chemie International Edition* **2020**, *59*, 19329–19337, DOI: 10.1002/anie.202007715.
(13) Leonardi, R.; Zhang, Y.-M.; Rock, C. O.; Jackowski, S. *Progress in Lipid Research* **2005**, *44*, 125–153, DOI: https://doi.org/10.1016/j.plipres.2005.04.001.
(14) Moreau, R. J. et al. *Journal of Medicinal Chemistry* **2018**, *61*, 3309–3324, DOI: 10.1021/acs.jmedchem.7b01691.
(15) Dang, M.; Song, J. *Protein Science* **2022**, *31*, 345–356, DOI: 10.1002/pro.4221.
(16) Zhou, R.; Zeng, R.; von Brunn, A.; Lei, J. *Molecular Biomedicine* **2020**, *1*, DOI: 10.1186/s43556-020-00001-4.
(17) Márquez-Moñino, M.; Santiveri, C.; de León, P.; Camero, S.; Campos-Olivas, R.; Jiménez, M.; Sáiz, M.; González, B.; Pérez-Cañadillas, J. *Structure* **2025**, *33*, 39–50, DOI: 10.1016/j.str.2024.10.025.
(18) Cléry, A.; Krepl, M.; Nguyen, C.; Moursy, A.; Jorjani, H.; Katsantoni, M.; Okoniewski, M.; Mittal, N.; Zavolan, M.; Sponer, J.; Sponer, J.; Allain, F.-T. *Nature Communications* **2021**, *12*, DOI: 10.1038/s41467-020-20481-w.
(19) De Silva, N.; Fargason, T.; Zhang, Z.; Wang, T.; Zhang, J. *Biochemistry* **2022**, *61*, 2922–2932, DOI: 10.1021/acs.biochem.2c00565.
(20) Das, S.; Krainer, A. *Molecular Cancer Research* **2014**, *12*, 1195–1204, DOI: 10.1158/1541-7786.MCR-14-0131.
(21) De Silva, N.; Lehman, N.; Fargason, T.; Paul, T.; Zhang, Z.; Zhang, J. *Nucleic Acids Research* **2024**, *52*, 4676–4690, DOI: 10.1093/nar/gkae213.

(22) Shimoni-Sebag, A.; Lebenthal-Loinger, I.; Zender, L.; Karni, R. *Carcinogenesis* **2013**, *34*, 2498–2504, DOI: 10.1093/carcin/bgt247.

(23) Anczuków, O.; Rosenberg, A.; Akerman, M.; Das, S.; Zhan, L.; Karni, R.; Muthuswamy, S.; Krainer, A. *Nature Structural and Molecular Biology* **2012**, *19*, 220–228, DOI: 10.1038/nsmb.2207.

(24) Guarnaccia, A. D.; Rose, K. L.; Wang, J.; Zhao, B.; Popay, T. M.; Wang, C. E.; Guerrazzi, K.; Hill, S.; Woodley, C. M.; Hansen, T. J.; Lorey, S. L.; Shaw, J. G.; Payne, W. G.; Weissmiller, A. M.; Olejniczak, E. T.; Fesik, S. W.; Liu, Q.; Tansey, W. P. *Cell Reports* **2021**, *34*, 108636, DOI: 10.1016/j.celrep.2020.108636.

(25) Thomas, L. R.; Adams, C. M.; Wang, J.; Weissmiller, A. M.; Creighton, J.; Lorey, S. L.; Liu, Q.; Fesik, S. W.; Eischen, C. M.; Tansey, W. P. *Proceedings of the National Academy of Sciences* **2019**, *116*, 25260–25268, DOI: 10.1073/pnas.1910391116.

(26) Ding, J.; Li, G.; Liu, H.; Liu, L.; Lin, Y.; Gao, J.; Zhou, G.; Shen, L.; Zhao, M.; Yu, Y.; Guo, W.; Hommel, U.; Ottl, J.; Blank, J.; Aubin, N.; Wei, Y.; He, H.; Sage, D. R.; Atadja, P. W.; Li, E.; Jain, R. K.; Tallarico, J. A.; Canham, S. M.; Chiang, Y.-L.; Wang, H. *ACS Chemical Biology* **2023**, *18*, 34–40, DOI: 10.1021/acschembio.2c00843.

(27) Chacón Simon, S.; Wang, F.; Thomas, L. R.; Phan, J.; Zhao, B.; Olejniczak, E. T.; Macdonald, J. D.; Shaw, J. G.; Schlund, C.; Payne, W.; Creighton, J.; Stauffer, S. R.; Waterson, A. G.; Tansey, W. P.; Fesik, S. W. *Journal of Medicinal Chemistry* **2020**, *63*, 4315–4333, DOI: 10.1021/acs.jmedchem.0c00224.

(28) Grebien, F. et al. *Nature Chemical Biology* **2015**, *11*, 571–578, DOI: 10.1038/nchembio.1859.

(29) Congreve, M.; Langmead, C. J.; Mason, J. S.; Marshall, F. H. Progress in structure based drug design for G protein-coupled receptors, 2011, DOI: 10.1021/jm200371q.

(30) Thomas, J.; Hergenrother, P. *Chemical Reviews* **2008**, *108*, 1171–1224, DOI: 10.1021/cr0681546.

(31) Campagne, S.; Jutzi, D.; Malard, F.; Matoga, M.; Romane, K.; Feldmuller, M.; Colombo, M.; Ruepp, M. D.; Allain, F. H. *Nature Communications* **2023**, *14*, DOI: 10.1038/s41467-023-40254-5.

(32) Wang, E.; Lu, S. X.; Pastore, A.; Chen, X.; Imig, J.; Chun-Wei Lee, S.; Hockemeyer, K.; Ghebrechristos, Y. E.; Yoshimi, A.; Inoue, D.; Ki, M.; Cho, H.; Bitner, L.; Kloetgen, A.; Lin, K.-T.; Uehara, T.; Owa, T.; Tibes, R.; Krainer, A. R.; Abdel-Wahab, O.; Aifantis, I. *Cancer Cell* **2019**, *35*, 369–384, DOI: 10.1016/j.ccell.2019.01.010.

(33) Tengs, T.; Kristoffersen, A. B.; Bachvaroff, T. R.; Jonassen, C. M. *Virology Journal* **2013**, *10*, 132, DOI: 10.1186/1743-422X-10-132.

(34) Childs-Disney, J. L.; Yang, X.; Gibaut, Q. M.; Tong, Y.; Batey, R. T.; Disney, M. D. *Nature Reviews Drug Discovery* **2022**, *21*, 736–762, DOI: 10.1038/s41573-022-00521-4.

(35) Sreeramulu, S. et al. *Angewandte Chemie - International Edition* **2021**, *60*, 19191–19200, DOI: 10.1002/anie.202103693.

(36) Harrar, Y.; Bellini, C.; Faure, J.-D. *Trends in Plant Science* **2001**, *6*, 426–431, DOI: 10.1016/S1360-1385(01)02044-1.

(37) Stebbins, J. L.; Zhang, Z.; Chen, J.; Wu, B.; Emdadi, A.; Williams, M. E.; Cashman, J.; Pellecchia, M. *Journal of Medicinal Chemistry* **2007**, *50*, 6607–6617, DOI: 10.1021/jm0707424.

(38) Palmioli, A.; Sperandeo, P.; Polissi, A.; Airoldi, C. *ChemBioChem* **2019**, *20*, 2911–2915, DOI: 10.1002/cbic.201900383.

(39) Siebs, E.; Shanina, E.; Kuhaudomlarp, S.; da Silva Figueiredo Celestino Gomes, P.; Fortin, C.; Seeberger, P.; Rognan, D.; Rademacher, C.; Imberty, A.; Titz, A. *ChemBioChem* **2022**, *23*, DOI: 10.1002/cbic.202100563.

(40) Gillon, E.; Varrot, A.; Imberty, A. In *Lectin Purification and Analysis*, Jun Hirabayashi, Ed.; Springer: 2020, pp 475–482, DOI: 10.1007/978-1-0716-0430-4{_}46.

(41) Shanina, E.; Siebs, E.; Zhang, H.; Varón Silva, D.; Joachim, I.; Titz, A.; Rademacher, C. *Glycobiology* **2021**, *31*, 159–165, DOI: 10.1093/glycob/cwaa057.

(42) Kuhaudomlarp, S.; Siebs, E.; Shanina, E.; Topin, J.; Joachim, I.; da Silva Figueiredo Celestino Gomes, P.; Varrot, A.; Rognan, D.; Rademacher, C.; Imberty, A.; Titz, A. *Angewandte Chemie - International Edition* **2021**, *60*, 8104–8114, DOI: 10.1002/anie.202013217.
(43) Leusmann, S.; Siebs, E.; Kuhaudomlarp, S.; Varrot, A.; Imberty, A.; Kuhn, B.; Lerner, C.; Grether, U.; Titz, A. *Angewandte Chemie - International Edition* **2025**, DOI: 10.1002/anie.202508864.
(44) Lingel, A.; Vulpetti, A.; Reinsperger, T.; Proudfoot, A.; Denay, R.; Frommlet, A.; Henry, C.; Hommel, U.; Gossert, A.; Luy, B.; Luy, B.; Frank, A. *Angewandte Chemie - International Edition* **2020**, *59*, 14809–14817, DOI: 10.1002/anie.202002463.
(45) Korn, S. M.; Lambertz, R.; Fürtig, B.; Hengesbach, M.; Löhr, F.; Richter, C.; Schwalbe, H.; Weigand, J. E.; Wöhnert, J.; Schlundt, A. *Biomolecular NMR Assignments* **2021**, *15*, 129–135, DOI: 10.1007/s12104-020-09995-y.
(46) Padroni, G.; Bikaki, M.; Novakovic, M.; Wolter, A. C.; Rüdisser, S. H.; Gossert, A. D.; Leitner, A.; Allain, F. H.-T. *Nucleic Acids Research* **2023**, *51*, 4555–4571, DOI: 10.1093/nar/gkad195.
(47) Altincekic, N. et al. *Frontiers in Molecular Biosciences* **2021**, *8*, DOI: 10.3389/fmolb.2021.653148.
(48) Gomori, G. In 1955, pp 138–146, DOI: 10.1016/0076-6879(55)01020-3.
(49) Rößler, P.; Ruckstuhl, M.; Löbbert, A.; Stühlinger, T.; Franchini, L. R.; Tsai, C.-J.; Lichtenecker, R.; Shrestha, B.; Rüdisser, S. H.; Konrat, R.; Schertler, G. F. X.; Gossert, A. D. *bioRxiv* **2024**, 2024.04.09.588766.
(50) Rüdisser, S. H.; Goldberg, N.; Ebert, M.-O.; Kovacs, H.; Gossert, A. D. *Journal of Biomolecular NMR* **2020**, *74*, 579–594, DOI: 10.1007/s10858-020-00325-x.
(51) Shaka, A.; Keeler, J.; Freeman, R. *Journal of Magnetic Resonance (1969)* **1983**, *53*, 313–340, DOI: 10.1016/0022-2364(83)90035-5.
(52) Widmer, A. Witnotp: A computer program for molecular modeling. Basel, 1997.
(53) Clark, M.; Cramer, R. D.; Van Opdenbosch, N. *Journal of Computational Chemistry* **1989**, *10*, 982–1012, DOI: 10.1002/jcc.540100804.
(54) Frisch, M. J. et al. Gaussian˜16 {R}evision {C}.01, 2016.
(55) Rüdisser, S.; Vangrevelinghe, E.; Maibaum, J. In *Fragment-based Drug Discovery Lessons and Outlook*; Wiley-VCH Verlag GmbH & Co. KGaA: 2016, pp 447–486, DOI: 10.1002/9783527683604.ch19.
(56) Dunning Thom H., J. *The Journal of Chemical Physics* **1989**, *90*, 1007–1023, DOI: 10.1063/1.456153.
(57) Zhao, Y.; Truhlar, D. G. *Theoretical Chemistry Accounts* **2008**, *120*, 215–241, DOI: 10.1007/s00214-007-0310-x.
(58) Marenich, A. V.; Cramer, C. J.; Truhlar, D. G. *Journal of Physical Chemistry B* **2009**, *113*, 6378–6396, DOI: 10.1021/jp810292n.
(59) Ditchfield, R. *Molecular Physics* **1974**, *27*, 789–807, DOI: 10.1080/00268977400100711.
(60) Wolinski, K.; Hinton, J. F.; Pulay, P. *Journal of the American Chemical Society* **1990**, *112*, 8251–8260, DOI: 10.1021/ja00179a005.
